# Identification of EnvC and Its Cognate Amidases as Novel Determinants of Intrinsic Resistance to Cationic Antimicrobial Peptides

**DOI:** 10.1128/AAC.02699-15

**Published:** 2016-03-25

**Authors:** Tamiko Oguri, Won-Sik Yeo, Taeok Bae, Hyunwoo Lee

**Affiliations:** a Center for Biomolecular Sciences, College of Pharmacy, University of Illinois at Chicago, Chicago, Illinois, USA; b Department of Biopharmaceutical Sciences, College of Pharmacy, University of Illinois at Chicago, Chicago, Illinois, USA; c Department of Microbiology and Immunology, Indiana University School of Medicine-Northwest, Gary, Indiana, USA

## Abstract

Cationic antimicrobial peptides (CAMPs) are an essential part of the innate immune system. Some Gram-negative enteric pathogens, such as Salmonella enterica, show intrinsic resistance to CAMPs. However, the molecular basis of intrinsic resistance is poorly understood, largely due to a lack of information about the genes involved. In this study, using a microarray-based genomic technique, we screened the Keio collection of 3,985 Escherichia coli mutants for altered susceptibility to human neutrophil peptide 1 (HNP-1) and identified *envC* and *zapB* as novel genetic determinants of intrinsic CAMP resistance. In CAMP killing assays, an E. coli Δ*envC*_Ec_ or Δ*zapB*_Ec_ mutant displayed a distinct profile of increased susceptibility to both LL-37 and HNP-1. Both mutants, however, displayed wild-type resistance to polymyxin B and human β-defensin 3 (HBD3), suggesting that the intrinsic resistance mediated by EnvC or ZapB is specific to certain CAMPs. A corresponding Salmonella Δ*envC*_Se_ mutant showed similarly increased CAMP susceptibility. The *envC* mutants of both E. coli and S. enterica displayed increased surface negativity and hydrophobicity, which partly explained the increased CAMP susceptibility. However, the Δ*envC*_Ec_ mutant, but not the Δ*envC*_Se_ mutant, was defective in outer membrane permeability, excluding this defect as a common factor contributing to the increased CAMP susceptibility. Animal experiments showed that the Salmonella Δ*envC*_Se_ mutant had attenuated virulence. Taken together, our results indicate that the role of *envC* in intrinsic CAMP resistance is likely conserved among Gram-negative enteric bacteria, demonstrate the importance of intrinsic CAMP resistance for full virulence of S. enterica, and provide insight into distinct mechanisms of action of CAMPs.

## INTRODUCTION

Antimicrobial peptides are an essential part of innate immune systems in vertebrates (1). In humans, about 100 antimicrobial peptides have been identified and reported to have antibacterial activity against Gram-negative and/or Gram-positive pathogens (2). Human neutrophils, in particular, produce two structurally distinct classes of antimicrobial peptides: an α-helical cathelicidin (LL-37) and β-sheet α-defensins (human neutrophil peptides 1 to 4 [HNP-1 to HNP-4]). Although structurally distinct, α-helical LL-37 and β-sheet HNP-1 (a representative α-defensin) share common properties, such as amphipathicity and cationicity, and both belong to a large family of cationic antimicrobial peptides (CAMPs). Due to their overall positive charges and amphipathic properties, CAMPs are thought to interact electrostatically with negatively charged molecules on the bacterial cell surface, e.g., lipopolysaccharides (LPS) in Gram-negative bacteria, and subsequently to target negatively charged cytoplasmic membranes to ultimately cause bacterial cell death (2).

However, several recent studies showed that following the initial interaction with lipopolysaccharides, CAMPs enter bacterial cells through distinct entry sites (3, 4) and/or bind specific receptor molecules on the cell wall and/or in the periplasm (5, 6). For example, LL-37 and another α-helical peptide, cecropin A, were shown to enter preferentially via septal sites in septating cells and via new cell division poles in nonseptating cells (3, 4). In both cases, cardiolipin, a negatively charged phospholipid, was suspected to attract CAMPs, because it is enriched in both septal sites and new cell division poles. Furthermore, LL-37 and some other α-helical CAMPs were shown to bind the lipoprotein Lpp in Escherichia coli (5) and its orthologue OprI in Pseudomonas aeruginosa (6). Such binding appears to promote the bactericidal action of those CAMPs (5, 6). It is currently unknown whether β-sheet peptides, such as HNP-1, also enter bacterial cells via preferential sites.

CAMP resistance in Salmonella enterica serovar Typhimurium has been studied extensively (7). In Salmonella, the PhoP/PhoQ and PmrA/PmrB two-component systems (TCS) mainly control inducible (or adaptive) resistance mechanisms to CAMPs. Each two-component system is comprised of a signal recognition sensor kinase (PhoQ and PmrB) and a cognate transcriptional regulator (PhoP and PmrA). Upon recognition of an inducing signal, the sensor kinase autophosphorylates and transfers phosphate to its partner DNA-binding transcriptional regulator, which controls the expression of target genes. PhoP/PhoQ is activated by low Mg^2+^, mildly acidic pH, and certain CAMPs (8), while PmrA/PmrB senses high Fe^3+^ or Al^3+^ and mildly acidic pH (9). PmrA-activated genes mostly mediate lipopolysaccharide modifications that reduce negative cell surface charges and confer resistance mainly to the cyclic lipopeptide polymyxin B (9). Although PhoP activates the PmrA/PmrB TCS (via PmrD) (10), it also controls genes involved in other resistance mechanisms for CAMPs, i.e., proteolytic degradation (11), peptide uptake and internal inactivation (12), and interference with the binding of CAMPs (13). As a result, PhoP-activated genes confer resistance to a broader spectrum of CAMPs, including polymyxin B, certain α-helical CAMPs (such as LL-37 and C18G) (14), and the β-sheet rabbit defensin NP-1 (15).

In contrast to the inducible resistance mechanisms for CAMPs, few intrinsic resistance mechanisms have been described for Gram-negative enteric bacteria (16). In particular, relative to α-helical LL-37, the β-sheet peptide HNP-1 exhibits much weaker antibacterial activity, requiring a much higher concentration to attain a level of bacterial killing similar to that by LL-37, suggesting that Gram-negative enteric pathogens are more intrinsically resistant to HNP-1. In this study, we attempted to uncover genes required for intrinsic resistance to HNP-1, and we identified two genes previously unknown to be involved in resistance to CAMPs: *envC* (*yibP*) and *zapB* (*yiiU*). Our results show that inactivation of *envC* causes multiple perturbations, including increased surface negativity and hydrophobicity, the combination of which renders cells hypersusceptible to HNP-1 and LL-37 but not to HBD-3 and polymyxin B. Furthermore, a Salmonella Δ*envC* mutant had attenuated virulence, indicating that intrinsic EnvC-mediated CAMP resistance is important for full virulence of S. enterica.

## MATERIALS AND METHODS

### Bacterial strains, growth conditions, and transductions.

All strains used in this study are listed in Table S1 in the supplemental material. The Keio collection of 3,985 E. coli single-gene deletion mutants was obtained from the laboratory of Hirotada Mori. E. coli BW25113, the parent strain for the construction of the Keio collection (17), and Salmonella enterica serovar Typhimurium 14028s (18) were used as the wild-type strains. Gene deletions were performed using the lambda Red system (19), and P1 and P22 transductions were conducted as described previously (20). All strains were grown in Luria-Bertani (LB) medium in 50-ml conical tubes or Erlenmeyer flasks at 37°C with shaking (200 rpm). Where necessary, appropriate antibiotics were added to the medium at the following concentrations for both E. coli and S. enterica: ampicillin, 100 μg/ml; kanamycin, 40 μg/ml; and streptomycin-spectinomycin, 10 μg/ml-50 μg/ml. E. coli JM109 and DH5α were used as host strains for cloning and preparation of plasmids.

### Genome-wide screen for mutants with altered HNP-1 susceptibility.

For application of the microarray-based genomic technique, i.e., monitoring of gene knockouts (MGK) (21), individual E. coli deletion mutants in the Keio collection were grown in 96-deep-well plates overnight at 37°C to an optical density at 600 nm (OD_600_) of about 1.3 in LB medium containing 40 μg/ml kanamycin, and then cultures were combined at similar ratios to make a pooled library. Cells were harvested by centrifugation, washed with fresh LB medium, and resuspended in LB medium supplemented with 15% glycerol. Aliquots containing about 1 × 10^9^ cells in 500 μl were frozen and stored at −80°C until use. The pooled Keio collection mutants were screened by MGK as described by Smith et al. (21). MGK simultaneously monitors the abundance of individual mutants in the pooled library and allows for rapid identification of mutants with altered fitness under a selective condition compared to a control condition. Briefly, the frozen stock of the pooled library was thawed at room temperature, and cells were harvested by centrifugation. After washing the cells twice with fresh LB medium, the pooled library was resuspended at ∼5 × 10^6^ cells/ml in LB medium and allowed to grow for two generations. Cells were then harvested by centrifugation, washed twice with 0.5% tryptone, and resuspended in 0.5% tryptone at ∼5 × 10^6^ cells/ml. The final cell suspension (450 μl) was mixed with HNP-1 (50 μl of a 500-μg/ml stock dissolved in 0.01% acetic acid) to a final concentration of 50 μg/ml; the same volume of 0.01% acetic acid without HNP-1 served as a control. After incubation for 1 h at 37°C with shaking, cells were harvested by centrifugation and plated onto LB agar. The next day, surviving cells were harvested and subjected to a second cycle of HNP-1 and control selections. Surviving cells harvested from the second selection, with and without HNP-1, were used for preparation of genomic DNAs, which were then used as templates for generation of microarray target DNAs (called MGK targets) as described by Smith et al. (21). The MGK targets of the HNP-1 selection and control were labeled with a fluorescent dye, either Alexa Fluor 555 or Alexa Fluor 647 (Life Technologies), and were then mixed in equal portions and hybridized to a custom microarray chip (CombiMatrix, Mukilteo, WA). Readouts from the scanned microarray images were normalized and used to calculate intensity ratios (values for the control/values for HNP-1 selection) for each probe. We classified probes with significant changes (≥2-fold) (control/HNP-1) as HNP-1-hypersusceptible probes. Details about the custom microarray chip design (selection of probe DNA sequences and negative-control probes), microarray hybridization, data acquisition/processing, and analysis can be found in our previously published paper (21).

### CAMP killing assay.

The following CAMPs were used in this study: HNP-1 (Bachem), polymyxin B sulfate (Sigma), and HBD-3 (AnaSpec). LL-37 was custom synthesized (Sigma) or purchased (AnaSpec). The amino acid sequences of CAMPs used in this study are shown in Fig. S1 in the supplemental material. MIC determination is not a reasonable assay for HNP-1 and other CAMPs due to the high cost and to deactivation in high-salt media and/or during incubation (22). To determine the susceptibility of wild-type and isogenic mutant strains to HNP-1, we used a killing assay in which the survival rates of cells were compared. Cells grown overnight in LB medium at 37°C with shaking at 200 rpm were diluted 100-fold in fresh LB medium and then grown to mid-exponential phase (OD_600_ of ∼0.5). Cells were harvested by centrifugation, washed with 0.5% tryptone three times, and resuspended in 0.5% tryptone at ∼2.5 × 10^6^ cells/ml. Forty-five microliters of resuspended cells was mixed with 5 μl of 10× HNP-1 in 96-well plates. Each 96-well plate was incubated on a Brinkmann Titermix instrument at 37°C with shaking (850 rpm), and 10-μl samples were taken and serially diluted in 90 μl phosphate-buffered saline (PBS) at the chosen time points. The number of surviving cells was determined by the spot plate method as described by Chen et al. (23). Percent survival was calculated as follows: % survival = (number of CFU of surviving cells/number of CFU of initial inoculum) × 100. The statistical significance of differences was calculated using unpaired, two-tailed Student's *t* test or one-way analysis of variance (ANOVA). Due to the potency differences that result from batch variation and daily preparation of CAMPs, only determinations with wild-type survival rates between 50 and 90% were accepted for comparison. Killing assays with other CAMPs and antibiotics were performed similarly.

### Construction of complementation plasmids.

To construct complementation plasmids, target genes (*envC*, *zapB*, and *yjgX*) with upstream native promoter regions were PCR amplified using the genomic DNA of E. coli BW25113 as the template. Primers used in this study are listed in Table S2 in the supplemental material. PCR-amplified DNA fragments were digested with appropriate restriction enzymes and cloned into the low-copy-number plasmid pCL1920 (24).

### Outer membrane permeability assay.

Outer membrane permeability was determined by measuring the influx of the cationic dye ethidium bromide or the neutral dye Nile red (25, 26). Cells were grown in LB medium, collected at mid-log phase (OD_600_ of ∼0.4), washed twice with assay buffer (50 mM KH_2_PO_4_, 137 mM NaCl, pH 7.0), and resuspended in the same buffer. Fluorescence was measured immediately after cells (final OD_600_ = 0.2) were mixed with ethidium bromide at a final concentration of 6 μM (excitation, 545 nm; and emission, 600 nm) or Nile red at a final concentration of 2 μM (excitation, 540 nm; and emission, 630 nm), using a SpectraMax M3 plate reader. Dye uptake assays were typically performed in the presence of the proton motive force inhibitor carbonyl cyanide-*m*-chlorophenylhydrazone (CCCP) at a final concentration of 10 μM. As noted previously by Murata et al. (26), dye uptake rates of different strains varied between experiments, but the dye uptake patterns of experiments were similar. Each experiment was repeated multiple times with similar results, and representative results are shown in Fig. 5A and in Fig. S4 in the supplemental material.

### Cytochrome *c* binding assay.

The cytochrome *c* binding assay was performed as described previously by Kristian et al. (27). Overnight cultures of both E. coli and S. enterica strains were diluted 100-fold in LB medium. After growing for 2 h, cells were harvested and washed twice in morpholinepropanesulfonic acid (MOPS) buffer (20 mM, pH 7.4), and the cell density was adjusted to a final OD_600_ of 7 in the same buffer. Cells were then mixed with cytochrome *c* at a final concentration of 0.5 mg/ml, incubated for 10 min at room temperature, and pelleted by centrifugation (18,213 × *g*, 6 min). Cytochrome *c* incubated without bacteria in the same buffer served as a control. The amount of cytochrome *c* in the supernatants was quantified at the absorption maximum of the prosthetic group (530 nm). The percentage of bound cytochrome *c* was calculated from three independent experiments performed in duplicate.

### Hexadecane adhesion assay.

Both E. coli and S. enterica cells from overnight cultures were diluted 100-fold in LB medium and were grown to mid-log phase (OD_600_ of ∼0.4). Cells of 5-ml cultures were harvested, washed twice with PBS, and then resuspended in 1 ml of PBS. From the cell suspension, 100 μl of cells was diluted 10-fold in PBS, and the OD_600_ was measured (*C*_0_). The remaining 900 μl of cells was mixed with 200 μl of hexadecane. After vortexing for 1 min, the mixture was kept at room temperature until the phases had separated. One hundred microliters of cells from the lower, aqueous phase was taken and diluted in 900 μl of PBS for measurement of the OD_600_ (*C*_H_). The percent hydrocarbon adherence of cells was calculated as follows: % hydrocarbon adherence = [(*C*_0_ − *C*_H_)/*C*_0_] × 100.

### Animal experiments.

The protocol for the animal experiments was prepared according to the guidelines of the National Institutes of Health and was reviewed and approved by the Committee on the Ethics of Animal Experiments of the Indiana University School of Medicine-Northwest (protocol number NW-36). Wild-type Salmonella and its isogenic mutant strains were grown overnight in LB broth at 37°C, pelleted, washed, and resuspended in sterile PBS. Respective bacterial cells were injected intraperitoneally into a group of 10 6- to 8-week-old C57BL/6 mice, and mouse survival was monitored for 2 weeks. The numbers of cells in the inocula used for mouse infection were determined by the spot plate method. Statistical analysis was performed using the log rank (Mantel-Cox) test.

### Statistical analysis.

All statistical analyses were performed using statistical modules in GraphPad Prism 6.

## RESULTS

### Identification of Escherichia coli mutants with altered HNP-1 susceptibility.

**FIG 1:**
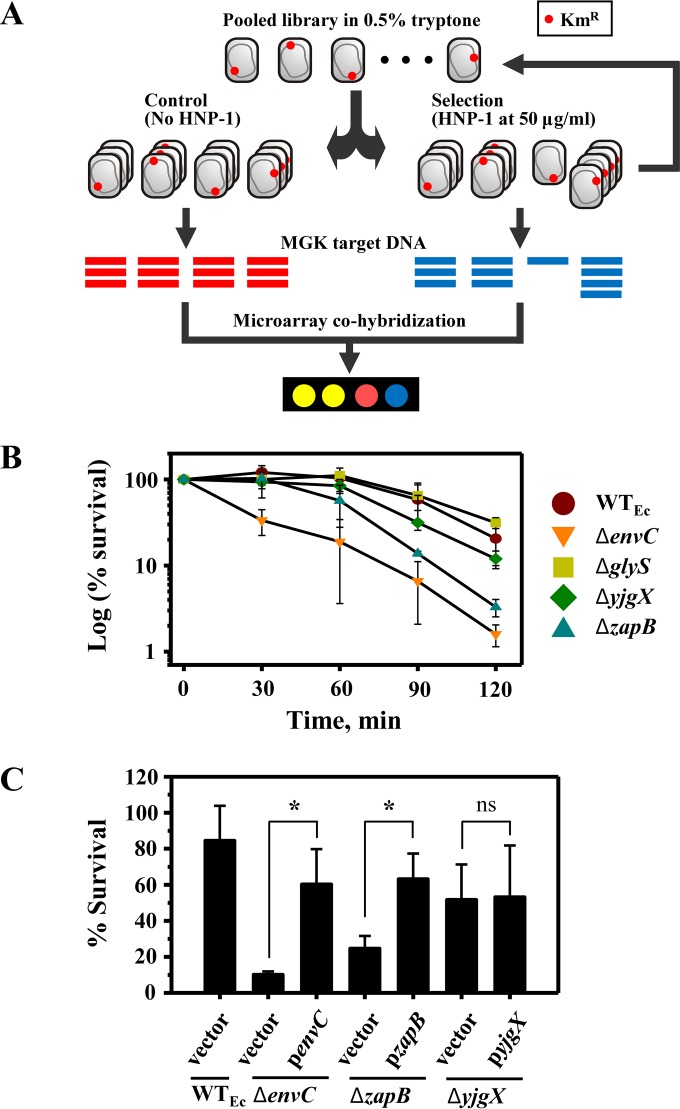
Identification of E. coli mutants with altered susceptibility to HNP-1. (A) Schematic diagram of MGK selection with the pooled library of Keio mutants, which were screened for mutants with altered susceptibility to HNP-1. Two cycles of MGK selection were performed to enrich the desired mutants. (B) Validation of individual mutants identified by MGK screening. Wild-type and mutant strains were grown to mid-log phase in LB medium. HNP-1 killing assays were performed for 2 h, during which the survival of tested strains was determined every 30 min. Data shown are the mean percentages of survival ± standard deviations (SD) for three independent experiments. The statistical significance of differences at the 2-h time point was determined by one-way ANOVA (for the wild type [WT_Ec_] versus the Δ*zapB* mutant, *P* ≤ 0.05; and for the WT_Ec_ versus the Δ*envC* mutant, *P* ≤ 0.005). (C) Genetic complementation of Δ*envC* and Δ*zapB* mutants restores wild-type HNP-1 susceptibility. The respective mutants were complemented with a plasmid carrying a wild-type copy of a corresponding gene with its own native promoter, while control strains harbored an empty vector. Complemented and control strains were examined for susceptibility to HNP-1 (100 μg/ml) by CAMP killing assays, and data are presented as the percent survival for three independent experiments (means and SD). Statistical significance was determined by two-tailed Student's *t* test. *, *P* ≤ 0.05; ns, not significant.

To identify the gene(s) involved in bacterial intrinsic resistance to human neutrophil peptide 1 (HNP-1), we screened the E. coli Keio collection of 3,985 nonessential deletion mutants by using a previously described microarray-based genomic method called monitoring of gene knockouts (MGK) (21), which simultaneously monitors the abundances of individual mutants within a library of mutants grown together as a population. The E. coli mutants were grown individually, pooled at equal starting ratios, and incubated for 1 h in the presence of HNP-1 at 50 μg/ml or with buffer alone as a control. After two cycles of HNP-1 selection (Fig. 1A), genomic DNAs were extracted and used as templates to generate MGK targets. The resulting MGK targets were fluorescently labeled with Alexa Fluor 555 (HNP-1 selection) and Alexa Fluor 647 (control) and mixed in equal portions for cohybridization onto a custom E. coli microarray chip. Readouts from the scanned microarray images were normalized and used to calculate intensity ratios (values for the control/values for HNP-1 selection) for the probes, each of which represents a mutant in the Keio collection. We classified probes with changes of ≥2-fold (control/HNP-1) as HNP-1-hypersusceptible probes. From this MGK screen, 34 mutants were putatively identified as being hypersusceptible to HNP-1 (see Table S3 in the supplemental material).

To validate the results of the MGK screen, individual mutations were transduced into a fresh isogenic wild-type E. coli strain by use of P1 bacteriophage and examined for HNP-1 susceptibility. HNP-1 killing assays were optimized such that the chosen concentration and time of exposure resulted in 50 to 99% survival of the wild-type strain. Most of the mutants initially identified as being hypersusceptible to HNP-1 did not exhibit consistent hypersusceptibility (data not shown), and two of these false-positive mutants, the Δ*glyS* and Δ*yjgX* mutants, served as controls (Fig. 1B). The validation ultimately yielded two hypersusceptible mutants (Δ*envC* and Δ*zapB*) (Fig. 1B).

To rule out the possibility of polar effects or secondary mutations, deletion mutants for genes adjacent to *envC* were tested, and genetic complementation was performed for determination of HNP-1 susceptibility. *envC* is the middle gene of a tricistronic operon (*gpmM-envC-yibQ*). A Δ*yibQ* mutant exhibited wild-type HNP-1 susceptibility, whereas a Δ*gpmM* mutant was as hypersusceptible to HNP-1 as the Δ*envC* mutant (see Fig. S2A in the supplemental material). In the Δ*gpmM* mutant from the Keio collection, the *gpmM* gene was deleted from the second codon to the seventh to last codon (17), and the *envC* gene was previously proposed to have its own promoter within this deleted region (28, 29). When complemented with the p*envC* plasmid, carrying a wild-type copy of *envC* with its ∼200-bp upstream region, both the Δ*gpmM* and Δ*envC* mutants had restored wild-type HNP-1 resistance, demonstrating that the HNP-1 hypersusceptibility of both mutants was solely due to a lack of *envC* expression (Fig. 1C; see Fig. S2A in the supplemental material). Complementation of the Δ*zapB* mutant with a plasmid carrying a wild-type copy of the corresponding deleted gene with its own promoter region restored HNP-1 susceptibility to wild-type levels (Fig. 1C). On the other hand, a false-positive Δ*yjgX* mutant strain, when complemented, did not show any change in HNP-1 susceptibility and served as a negative control (Fig. 1C). Taken together, these results establish the causal relationship between the individual deletions of *envC* and *zapB* and altered HNP-1 susceptibility.

### Δ*envC* and Δ*zapB* mutants exhibit distinct profiles of susceptibility to different CAMPs.

**FIG 2:**
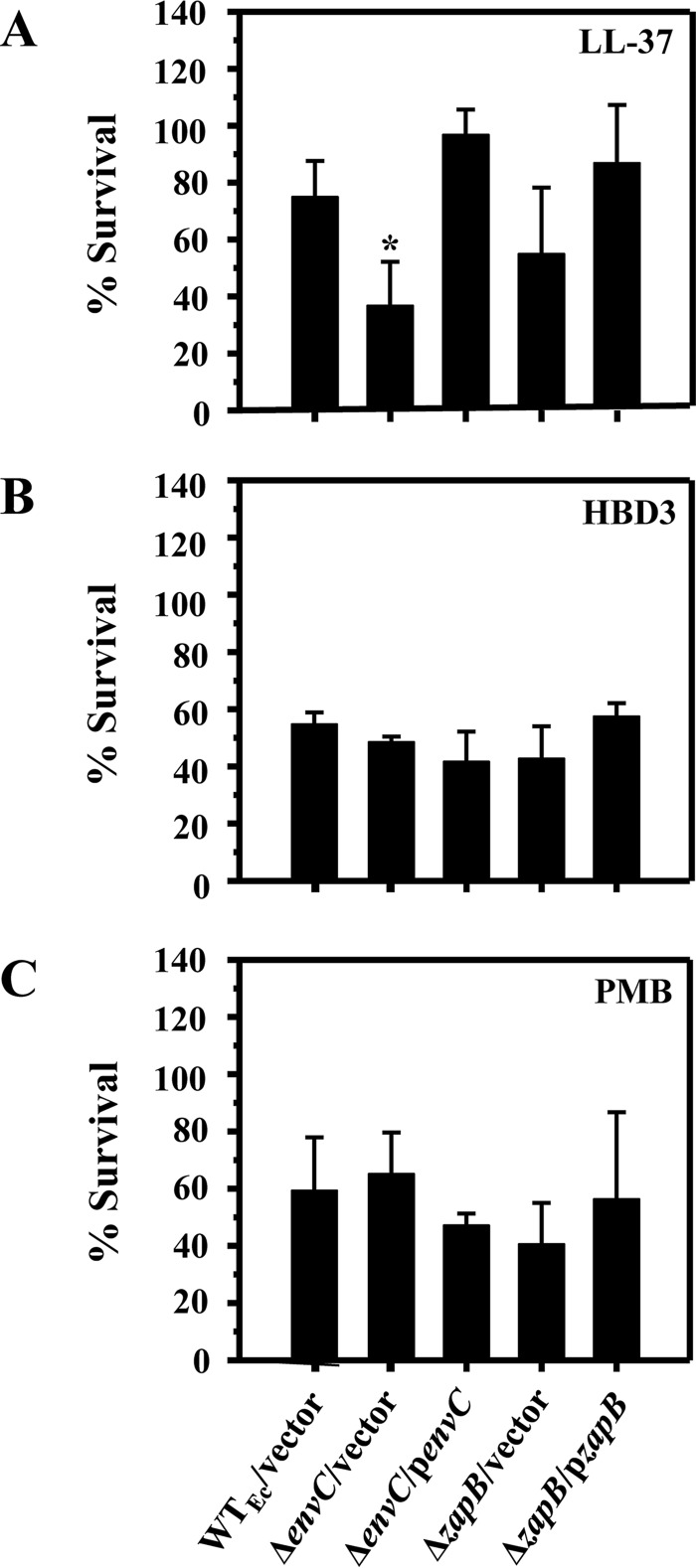
Susceptibility profiles of Δ*envC* and Δ*zapB* mutants for different CAMPs. (A) LL-37 at 5 μg/ml. (B) HBD3 at 5 μg/ml. (C) Polymyxin B (PMB) at 0.025 μg/ml. Killing assays were performed similarly to the description in the legend to Fig. 1, except that the assay mixture was incubated for 15 min. Data shown are the means and SD for three independent experiments. Statistical significance was determined by one-way ANOVA (WT_Ec_/vector versus Δ*envC* mutant/vector). *, *P* ≤ 0.05.

To determine whether the *envC* and *zapB* genes are involved in resistance to other CAMPs, we examined the susceptibility of the corresponding deletion mutants to the β-sheet peptide human β-defensin 3 (HBD3), the α-helical peptide LL-37, or the cyclic lipopeptide polymyxin B (Fig. 2; see Fig. S1 in the supplemental material for the amino acid sequences of these peptides). As performed with HNP-1, the killing assay conditions were optimized for each peptide such that the chosen concentration and time of exposure resulted in 50 to 99% survival of the wild-type strain.

The susceptibility profiles of the Δ*envC* and Δ*zapB* mutants against LL-37 were similar to those observed with HNP-1 (compare Fig. 2A with Fig. 1B; also see Table S4 in the supplemental material for MICs of HNP-1 and LL-37 against the Δ*envC* mutant). Despite the marginal (statistically insignificant) difference of the Δ*zapB* mutant compared to the wild type shown in Fig. 2A, a consistent difference was observed in LL-37 killing assays with extended incubations (data not shown). Unexpectedly, both the Δ*envC* and Δ*zapB* mutants displayed wild-type susceptibilities to both HBD3 and polymyxin B (Fig. 2B and C). These results show that Δ*envC* and Δ*zapB* mutants are not hypersusceptible to all CAMPs.

### The CAMP hypersusceptibility of the Δ*envC* mutant is due to a loss of amidase activity.

Both EnvC and ZapB are known components of the cell divisome complex, and both localize to septal sites (30–32). ZapB stabilizes the early divisome by interacting cooperatively with other proteins, including EnvC; therefore, *zapB* deletion is likely to affect the subsequent recruitment and activity of EnvC (33). Since the increased CAMP susceptibility of the Δ*zapB* mutant was likely due to a secondary effect of *zapB* deletion on EnvC, our follow-up study was focused on the Δ*envC* mutant.

**FIG 3:**
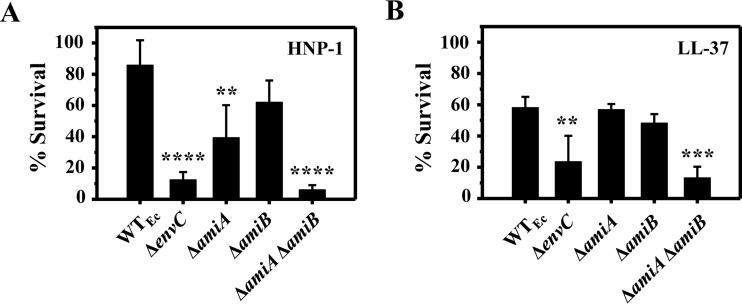
CAMP susceptibilities of *amiA* and *amiB* amidase mutants. (A) HNP-1 at 100 μg/ml. (B) LL-37 at 5 μg/ml. CAMP assays were performed similarly to the description in the legend to Fig. 1. Data shown are means and SD for three independent experiments. Statistical significance was determined by one-way ANOVA (**, *P* < 0.01; ***, *P* < 0.005; and ****, *P* < 0.001).

The *envC* gene encodes an accessory protein required for the activity of both AmiA and AmiB, which are *N*-acetylmuramoyl-l-alanine amidases that cleave between the stem peptide and *N*-acetylmuramic acid moieties of peptidoglycan during the early stages of cell division; however, its deletion causes only a minor defect in cell division (31). To determine whether the functional role of EnvC is linked to CAMP resistance, single and double amidase mutants were constructed and tested for HNP-1 susceptibility. In their 3′ regions, the *amiA* and *amiB* genes contain promoters for respective downstream genes (see Fig. S2B in the supplemental material) (34, 35), and to avoid polar effects, these promoter regions were kept intact in *amiA* and *amiB* deletion mutants (see Tables S1 and S2). In addition, deletion mutants of the downstream genes showed wild-type susceptibility to both HNP-1 and LL-37 (see Fig. S2B). Neither of the single deletion mutants (Δ*amiA* and Δ*amiB*) was as susceptible to HNP-1 as the Δ*envC* mutant (Fig. 3A), although the Δ*amiA* mutant exhibited a slightly increased HNP-1 susceptibility compared to that of the wild type. However, a Δ*amiA* Δ*amiB* double mutant was as susceptible to both HNP-1 and LL-37 as the Δ*envC* mutant (Fig. 3A and B). These results indicate that the increased CAMP susceptibility of the Δ*envC* mutant was due to the loss of amidase (both AmiA and AmiB) activity.

### The role of *envC* in intrinsic CAMP resistance is conserved in Salmonella enterica, another Gram-negative enteric bacterium.

S. enterica serovar Typhimurium has been the model Gram-negative enteric pathogen for studying resistance mechanisms for CAMPs (7). To determine whether the role of EnvC is also conserved in Salmonella, we constructed and tested an orthologous Salmonella Δ*envC* (Δ*envC*_Se_) mutant for susceptibility to CAMPs. Two Salmonella mutant strains served as controls: a Δ*phoP*_Se_ mutant that is hypersusceptible to CAMPs, such as α-helical magainin 2 and polymyxin B, as well as to other classes of antibiotics, such as vancomycin (36), and a Δ*pmrAB*_Se_ mutant that is known to be exclusively hypersusceptible to polymyxin B (9). Since the wild-type S. enterica strain is intrinsically more resistant than E. coli to HNP-1, CAMP killing assays were conducted with a higher concentration of HNP-1 (250 μg/ml).

**FIG 4:**
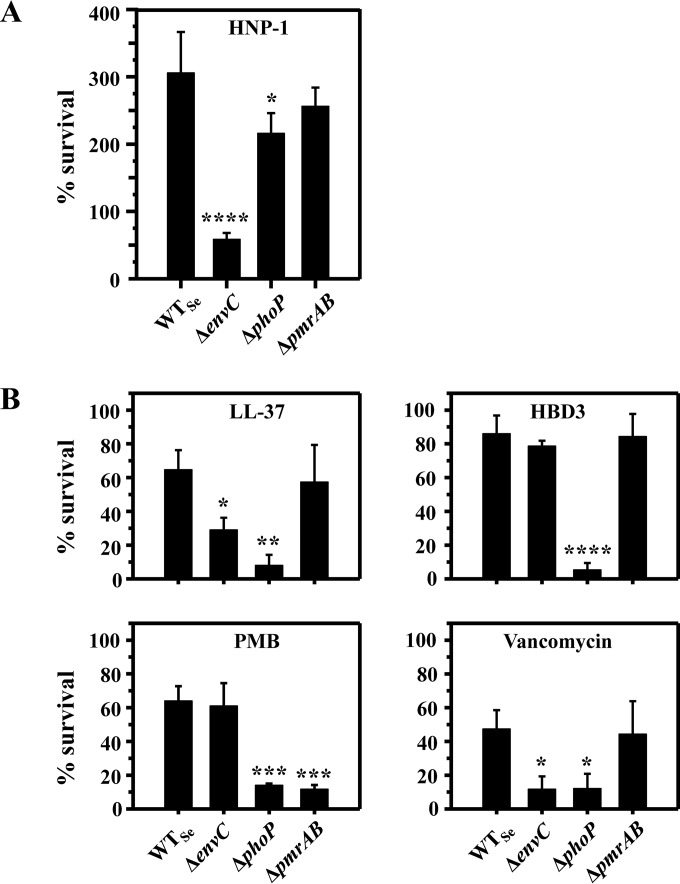
The Salmonella Δ*envC*_Se_ mutant exhibits increased CAMP susceptibility. (A) HNP-1 at 250 μg/ml. (B) Killing assays were performed similarly to the description in the legend to Fig. 1, with vancomycin at 500 μg/ml, LL-37 at 5 μg/ml, HBD3 at 5 μg/ml, or polymyxin B (PMB) at 0.1 μg/ml. Vancomycin was incubated for 1 h, and other CAMPs were incubated for 15 min. Data shown are means and SD for three independent experiments. Statistical significance was determined by one-way ANOVA (*, *P* < 0.05; **, *P* < 0.01; ***, *P* < 0.005; and ****, *P* < 0.001).

As expected, the Δ*phoP*_Se_ mutant was marginally susceptible to HNP-1 (Fig. 4A) and was highly susceptible to LL-37, polymyxin B, and vancomycin (Fig. 4B) (36), whereas the Δ*pmrAB*_Se_ mutant was hypersusceptible only to polymyxin B (Fig. 4A and B). The susceptibility of Δ*phoP*_Se_ and Δ*pmrAB*_Se_ strains to HBD3 has not been reported before; the Δ*phoP*_Se_ mutant exhibited highly increased susceptibility to HBD3, but the Δ*pmrAB*_Se_ mutant displayed wild-type susceptibility. The CAMP susceptibility trends of the Salmonella Δ*envC*_Se_ strain (Fig. 4) were very similar to those of the E. coli Δ*envC*_Ec_ strain (Fig. 1B and 2), i.e., hypersusceptibility to HNP-1 and LL-37 but not to HBD3 and polymyxin B. These results demonstrate that the role of EnvC in CAMP resistance is conserved in Salmonella, and likely in other related Gram-negative enteric bacteria.

### EnvC does not degrade CAMP, and outer membrane permeability does not explain the CAMP hypersusceptibility of the *envC* mutant.

To further our understanding of how mutation of *envC* and the consequent inactivation of the amidases AmiA and AmiB result in CAMP hypersusceptibility, we overexpressed and purified the EnvC protein as well as AmiA and AmiB. Since EnvC alone was previously shown to cleave the protein β-casein (37), we examined whether EnvC cleaves HNP-1. The EnvC protein in combination with either amidase (AmiA or AmiB) was able to cleave the peptidoglycan (see Fig. S3C in the supplemental material). However, it did not show any proteolytic activity against HNP-1, even in combination with its cognate amidases (see Fig. S3D), suggesting that the CAMP hypersusceptibility of the *envC* mutant was likely due to other cell surface changes caused by the lack of EnvC (and the loss of amidase activity), not to a direct action on the CAMP itself.

**FIG 5:**
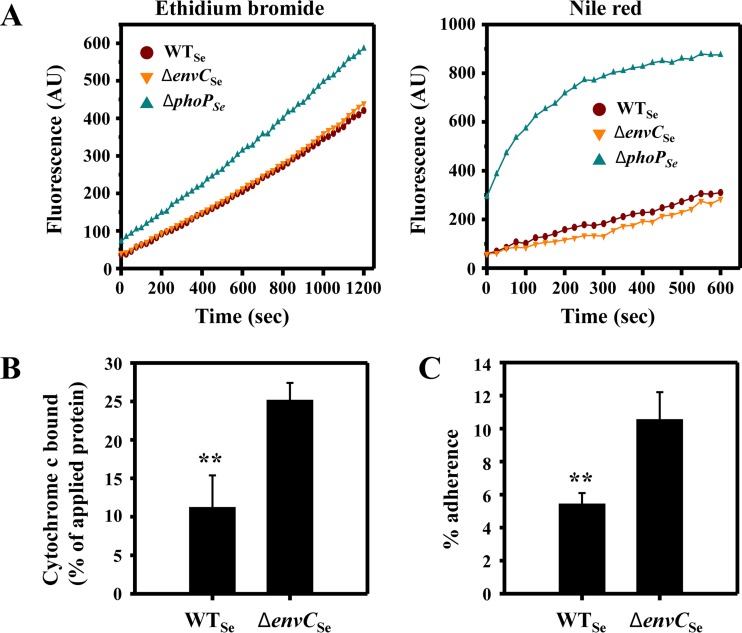
The Salmonella Δ*envC*_Se_ mutant exhibits wild-type outer membrane permeability but increased surface negativity and hydrophobicity. (A) Outer membrane permeability toward the cationic dye ethidium bromide or the neutral dye Nile red. Cells grown to mid-log phase in LB medium were harvested and resuspended in buffer to an OD_600_ of 4. After the addition of dye to the cell resuspension, the fluorescence was measured immediately and over time. Data shown are representative of more than three independent experiments with similar results. AU, arbitrary units. (B) Surface charges of the Salmonella wild-type and Δ*envC*_Se_ mutant strains were examined by a cytochrome *c* binding assay. Data shown are means and SD for three independent experiments. (C) Hydrophobicities of cell surfaces of the Salmonella wild-type and Δ*envC*_Se_ mutant strains were examined by a hexadecane adhesion assay. Data shown are means and SD for three independent experiments. Statistical significance was determined by two-tailed Student's *t* test (**, *P* < 0.01).

The Δ*envC*_Ec_ mutant has previously been shown to be hypersusceptible to many antibiotics with different modes of action (38), and we observed that the Salmonella Δ*envC*_Se_ mutant displayed increased susceptibility to the cell wall-targeting antibiotic vancomycin (Fig. 4B). This drug has a high molecular mass (1,450 Da), and increased sensitivity of the mutant could be an indication of increased outer membrane permeability (39). Therefore, we tested the outer membrane permeability of wild-type and Δ*envC* cells by measuring the uptake rates of two fluorescent dyes, namely, cationic ethidium bromide and neutral Nile red. The dye uptake assay was performed in the presence of the proton motive force inhibitor CCCP to eliminate the antagonizing effect of multidrug efflux pumps (25, 26). The Δ*phoP* mutant, known for increased outer membrane permeability, served as a control (26). Compared with wild-type E. coli, the Δ*envC*_Ec_ mutant exhibited extremely high baseline outer membrane permeability to both ethidium bromide and Nile red (see Fig. S4 in the supplemental material), and it displayed slightly higher dye uptake rates early in the assay (up to ∼100 s), especially for ethidium bromide. After the initial surge of ethidium bromide uptake, the uptake rate of the Δ*envC*_Ec_ mutant became similar to that of wild-type E. coli. We suspected that this biphasic pattern of ethidium bromide uptake might be due to the presence of dead cells and cell debris. Indeed, this was supported by the presence of many ghost cells and cell debris revealed in electro-microscopic images of the Δ*envC*_Ec_ mutant (see Fig. S5 in the supplemental material). In marked contrast, the orthologous Salmonella Δ*envC*_Se_ mutant displayed wild-type levels of outer membrane permeability to both ethidium bromide and Nile red (Fig. 5A). The control Δ*phoP* strains of both E. coli and S. enterica showed more outer membrane permeability than the respective wild-type strains (Fig. 5A; see Fig. S4). Based on these results, we concluded that the outer membrane permeability defect was not a main contributing factor to the increased CAMP susceptibility of the Δ*envC* mutant.

### Inactivation of *envC* does not alter LPS length but results in increased surface charges and hydrophobicity.

Mutations causing the production of truncated lipooligosaccharides or the loss of the LPS O antigen result in increased susceptibility to certain CAMPs, such as human α-defensin 5 (HD-5) and LL-37 (40, 41). However, we confirmed that both E. coli and Salmonella Δ*envC* mutants produced wild-type LPS (see Fig. S6A in the supplemental material), excluding the linkage of *envC* deletion to aberrant LPS production.

Susceptibility to CAMPs can also be affected by surface charges and hydrophobicity of bacterial cells (7). To determine whether the surface properties of the Δ*envC*_Se_ mutant were altered, we examined surface charges by measuring the binding of the cationic protein cytochrome *c* to cells (27, 42) and determined surface hydrophobicity by cell adherence to hexadecane (43, 44). The Δ*envC*_Se_ mutant cells bound to cytochrome *c* about 2.5-fold more than wild-type cells did (Fig. 5B), and they adhered to hexadecane ∼2-fold more than the wild-type Salmonella cells did (Fig. 5C), indicating increased negative charges and hydrophobicity on the surface of the Δ*envC*_Se_ mutant. Similar cell surface alterations were also observed with the E. coli Δ*envC*_Ec_ mutant (see Fig. S7A and B in the supplemental material). These results suggested that the *envC* knockout caused changes in the composition of the cell envelope. Consistently, the examination of membrane protein profiles by two-dimensional gel electrophoresis revealed differential levels of some membrane proteins in the Δ*envC*_Ec_ mutant compared with the wild type (see Fig. S8). Taken together, the results indicate that the altered cell surface properties observed with the Δ*envC* mutants appear to be due in part to changes in membrane protein profiles and are expected to increase the binding of cationic, amphipathic antimicrobial peptides to Δ*envC* cells. Based on these results, we concluded that cell surface alterations contribute at least partly to the Δ*envC* hypersusceptibility to CAMPs.

### The Salmonella Δ*envC*_Se_ mutant has attenuated virulence.

**FIG 6:**
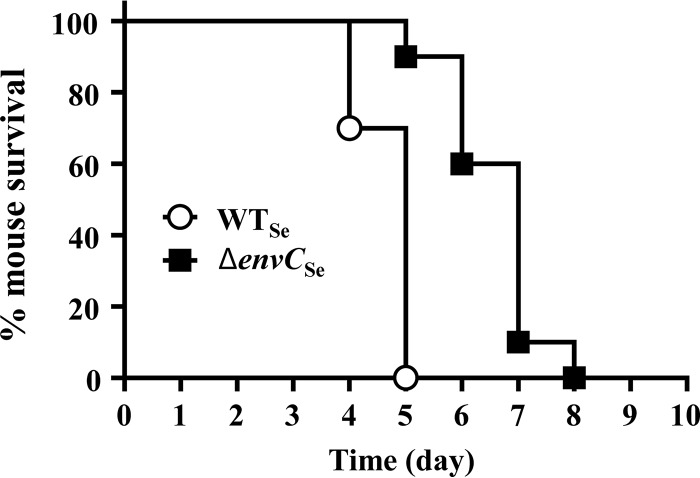
The Salmonella Δ*envC*_Se_ mutant displays attenuated virulence. Groups of 10 C57BL/6 mice were inoculated intraperitoneally with the Salmonella 14028s wild-type and Δ*envC*_Se_ mutant strains at a dose of 2 × 10^3^ cells. Statistical significance was determined by the log rank (Mantel-Cox) test (for the WT_Se_ versus the Δ*envC*_Se_ mutant, *P* < 0.0001).

The Δ*envC* mutant was found to be the most hypersusceptible to the major CAMPs produced by human neutrophils (HNP-1 and LL-37), with no appreciable growth defect *in vitro*. To determine the *in vivo* significance of this finding, we examined the virulence of the Δ*envC*_Se_ mutant in the mouse model of Salmonella infection. Mice were inoculated intraperitoneally with ∼2 × 10^3^ cells of the wild-type or Δ*envC*_Se_ strain. Mice infected with the Δ*envC*_Se_ mutant showed a significantly higher survival rate (*P* < 0.0001) than that of mice infected with wild-type Salmonella (Fig. 6), demonstrating that the role of EnvC, including intrinsic CAMP resistance, is important for full virulence of Salmonella.

## DISCUSSION

Intrinsic resistance to host CAMPs, such as HNP-1, is a crucial factor for bacterial pathogen survival in host environments, yet little is known about the genes required for the intrinsic resistance to HNP-1 in Gram-negative bacteria. In this study, we screened the Keio collection of 3,985 E. coli mutants and identified two genes (*envC* and *zapB*) previously unknown for their roles in intrinsic resistance to CAMPs (Fig. 1 and 2). Since ZapB is necessary for stabilization of the FtsZ ring at the septum and the subsequent recruitment of several proteins, including EnvC (32), it is likely that the hypersusceptibility phenotypes of the Δ*envC* and Δ*zapB* mutants were due to impairment in this shared pathway. Several other genes that are involved in Z-ring and septum formation are essential for viability (45); therefore, the corresponding mutants are not available in the Keio collection and would not appear in our screen (17). In a recent study, Moser et al. also screened the Keio collection for mutants with increased sensitivity to HD-5, and they identified mutants with a defect in either LPS biosynthesis or membrane integrity (46). However, the Δ*envC*_Ec_ mutant was reported to exhibit very weak, if any, hypersusceptibility to HD-5, and the Δ*zapB* mutant was not retrieved. This result is somewhat similar to our observation that both E. coli and S. enterica Δ*envC* mutants display wild-type susceptibility to HBD3 (Fig. 2 and 4) and may suggest that the mechanism of action of HD-5 is similar to that of HBD3 but distinct from that of HNP-1. In another study, Weatherspoon-Griffin et al. screened a collection of ∼1,400 Salmonella single-gene deletion mutants for those with increased protamine susceptibility (47), and they identified a Δ*tatC* mutant defective in the twin-arginine transport (Tat) system (48) and Δ*amiA* and Δ*amiC* mutants that lack amidases translocated by the Tat system (49). Though single or double amidase mutants were hypersusceptible to the linear peptide protamine and the α-helical peptides magainin 2 and melittin, they were not hypersusceptible to either polymyxin B or HNP-1, suggesting that the mechanism of action of HNP-1 is distinct from that of protamine, magainin 2, or melittin (47).

Most, if not all, CAMPs, including defensins, were thought to target bacterial cell membranes (1, 50–53). However, increasing evidence suggests that many CAMPs may have additional, more specific targets (50). A study by de Leeuw et al. showed that the bactericidal activity of HNP-1 does not correlate with membrane leakage in Staphylococcus aureus but instead HNP-1 binds the peptidoglycan building block lipid II *in vitro* (54). (We refer to lipid II molecules as those both newly synthesized and incorporated into peptidoglycan here.) It is also noteworthy that LL-37 was shown to enter the cell via new cell division poles and/or septal sites, where newly synthesized lipid II molecules are likely to be enriched (4). Since EnvC forms an active amidase with either AmiA or AmiB that cleaves the amide bond between the d-alanine residue of the stem peptide and *N*-acetylmuramic acid in the peptidoglycan (31), the peptidoglycan of the Δ*envC* mutant is expected to be packed more densely with (un)cross-linked lipid II molecules (55). Though the direct binding of LL-37 to lipid II remains to be determined, it is highly probable that enriched lipid II molecules in the Δ*envC* mutant attract more lipid II-targeting CAMPs.

While our results align well with the notion that both HNP-1 and LL-37 may target lipid II molecules, the distinct susceptibility patterns of the Δ*envC* mutant and its cognate amidase mutants to different CAMPs imply that HNP-1 and LL-37 may particularly target certain species of lipid II molecules (Fig. 3 and 4). Interestingly, though the AmiA and AmiB amidases are both activated by EnvC and can cleave lipid II molecules with either tetra- or penta-stem peptides (31, 56), AmiA has a stronger substrate preference for lipid II molecules with penta-stem peptides (56). We consistently observed the Δ*amiA* mutant exhibiting slightly increased susceptibility to HNP-1 (Fig. 3A) compared with the Δ*amiB* mutant, which exhibited wild-type HNP-1 susceptibility. This result suggests that HNP-1 may bind lipid II molecules with penta-stem peptides, while LL-37 preferentially binds lipid II molecules with tetra-stem peptides. This notion is further corroborated by additional observations with the Δ*nlpD* and Δ*amiC* mutants. E. coli harbors a third amidase, AmiC, which is activated by its cognate accessory protein NlpD (31). AmiC cleaves the same amide bond in peptidoglycan as that cleaved by AmiA and AmiB, but it preferentially cleaves lipid II molecules with tetra-stem peptides over those with penta-stem peptides (31, 56). Consistently, both Δ*amiC* and Δ*nlpD* mutants displayed wild-type HNP-1 susceptibility, but they exhibited increased susceptibility to LL-37 compared with that of the wild type (see Fig. S9 in the supplemental material). Nevertheless, the preferential binding of these CAMPs to lipid II molecules with stem peptides of different lengths remains to be determined.

In addition to potential enrichment of lipid II molecules in the Δ*envC* mutant, there appears to be an additional factor(s) involved in the Δ*envC* hypersusceptibility to CAMPs. Based on the localized entry of LL-37 into cells via septal sites, Sochacki et al. proposed cardiolipin, a negatively charged phospholipid that is normally enriched in septal sites of dividing cells, as a putative target of LL-37 (4). Interestingly, an earlier study by Michel et al. reported a 2-fold increase in cardiolipin content in the *E. coli envC* mutant (57). The results of these two studies may partly explain the overall increase in surface negativity and hydrophobicity and the consequent CAMP hypersusceptibility of the Δ*envC* mutant observed in this study (Fig. 1C, 2A, and 4).

Recently, Ercoli et al. reported that *envC* knockout alters the outer membrane and periplasmic protein compositions in Haemophilus influenzae (58), which is consistent with our observations with the E. coli Δ*envC* mutant (see Fig. S8 in the supplemental material). While it is clear that functional EnvC is required for proper localization of membrane/periplasmic proteins, it is unclear at this point whether and how the altered membrane protein composition contributes to the CAMP hypersusceptibility of the Δ*envC* mutant. It appears that the effect of *envC* inactivation in E. coli is limited to certain membrane/periplasmic proteins (see Table S5), since the Δ*envC*_Ec_ mutant exhibited no defect in LPS transport (see Fig. S6B). The contribution of the altered membrane/periplasmic protein compositions to the CAMP hypersusceptibility of the Δ*envC* mutant requires further examination.

Alternatively, or in addition, it is conceivable that anionic stem peptides, which are cleaved from lipid II by amidases (EnvC plus AmiA and EnvC plus AmiB), may directly bind and interfere with CAMPs, thereby conferring CAMP resistance. In E. coli, a negatively charged tetra-stem peptide (l-alanine-d-glutamate-*meso*-diaminopimelate-d-alanine) and its dimer (and perhaps trimer) are released/recycled during growth (59–61). In this scenario, anionic stem peptides are expected to play Jekyll and Hyde-type roles: those attached to lipid II are susceptibility determinants, and those liberated by amidases are resistance determinants.

The differences in outer membrane permeability we observed for the E. coli Δ*envC*_Ec_ mutant versus the Salmonella Δ*envC*_Se_ mutant (Fig. 5A; see Fig. S7 in the supplemental material) may have been due to strain- or species-specific differences and underscore the importance of validation in other related bacterial species. Our data clearly demonstrate that the CAMP hypersusceptibility of the Salmonella Δ*envC*_Se_ mutant is not due to defective outer membrane permeability (Fig. 5). In this regard, previous studies reporting the E. coli Δ*envC*_Ec_ mutant to be hypersusceptible to several antibiotics (38, 62) may need to be interpreted cautiously, because such phenotypes may not simply be due to a defect in outer membrane permeability.

Although it is significant, EnvC-mediated CAMP resistance may not be the only cause for the attenuated virulence of the Salmonella Δ*envC*_Se_ mutant. Because the Δ*envC*_Se_ mutant has an altered composition of membrane proteins, and likely of peptidoglycan, components that are recognized by the host immune system (63–65), it is reasonable to think that the Δ*envC*_Se_ mutant elicits an inflammatory response distinct from that to wild-type Salmonella. This altered host immune response may negatively influence the survival of the Δ*envC*_Se_ mutant in the host.

In conclusion, this study identifies the *envC* gene as a novel determinant of CAMP resistance, suggesting that intact cell division is critical for intrinsic resistance to certain CAMPs, such as HNP-1 and LL-37. Our data indicate that inactivation of *envC* causes multiple cellular perturbations, including increased surface negativity and hydrophobicity, the combination of which creates a microenvironment favorable for entry and subsequent action of CAMPs. Our results, together with two recent studies reporting the essentiality of *envC* in Pseudomonas aeruginosa (66) and in virulence of H. influenzae (58), suggest that the functional role of EnvC is conserved in a wide range of bacterial pathogens and support the proposition that compounds that inhibit the function of EnvC or its cognate amidases may serve as new antibacterial or antivirulence agents.

## Supplementary Material

Supplemental material
