# Supplementary material for: Identification of EnvC and Its Cognate Amidases as Novel Determinants of Intrinsic Resistance to Cationic Antimicrobial Peptides
Source: Antimicrob Agents Chemother. 2016 Mar 25;60(4):2222–31. doi: 10.1128/AAC.02699-15 (PMC4808223; doi:10.1128/AAC.02699-15)
Supplement: Supplemental material [file supp_60_4_2222__index.html]

Identification of EnvC and Its Cognate Amidases as Novel Determinants of Intrinsic Resistance to Cationic Antimicrobial Peptides — Supplemental material 

# Identification of EnvC and Its Cognate Amidases as Novel Determinants of Intrinsic Resistance to Cationic Antimicrobial Peptides

## Supplemental material

- Supplemental file 1 -

  Supplemental Materials and Methods, Tables S1-S5, and Fig. S1-S9

  PDF, 1.1M
