# Supplementary material for: Identification of EnvC and Its Cognate Amidases as Novel Determinants of Intrinsic Resistance to Cationic Antimicrobial Peptides
Source: Antimicrob Agents Chemother. 2016 Mar 25;60(4):2222–31. doi: 10.1128/AAC.02699-15 (PMC4808223; doi:10.1128/AAC.02699-15)
Supplement: Supplemental material [file AAC.02699-15_zac004165025so1.pdf]

SUPPLEMENTAL MATERIAL

Supplemental Methods

TABLE S1 Strains and plasmids used in this study

TABLE S2 Primers used in this study

TABLE S3 Mutants identified as putatively hypersusceptible or hyper-resistant to HNP-1

TABLE S4 MICs of HNP-1 and LL-37 against Δ*envC*

TABLE S5 Proteins whose expression level is altered in the Δ*envC* mutant relative to WT_{Ec}

FIG S1 Cationic antimicrobial peptides used in this study

FIG S2 The neighboring genes of *envC*, *amiA*, *amiB*, and *nlpD* are not involved in CAMP resistance

FIG S3 EnvC and its cognate amidases do not degrade HNP-1.

FIG S4 The *E. coli* Δ*envC* mutant exhibits altered outer membrane permeability

FIG S5 Microscopic examination of the *E. coli* wild-type and Δ*envC* strains

FIG S6 Inactivation of *envC* does not alter the length and distribution of LPS

FIG S7 The *E. coli* Δ*envC* mutant exhibits altered surface properties

FIG S8 Membrane and periplasmic protein profiles of the *E. coli* wild-type and Δ*envC* strains

FIG S9 The *E. coli* Δ*nlpD* and Δ*amiC* mutants are hypersusceptible to LL-37, but not to HNP-1

Supplemental References

Supplemental Methods

Determination of minimal inhibitory concentration (MIC) of antimicrobial peptides. MICs of HNP-1 and LL-37 were determined using the protocol of Turner *et al.* (1). This protocol uses "refined Mueller-Hinton broth (MHB)", instead of standard MHB (BBL 211443; Becton Dickinson), in which antimicrobial peptides such as PG-1 and LL-37 precipitate. Refined MHB was prepared by filtering standard MHB through a series of three Sep-Pak Aminopropyl (NH2) Plus columns (WAT020535; Waters). Bacterial strains were grown overnight in refined MHB and diluted 100-fold in fresh refined MHB. After grown to mid-log phase, cell density was adjusted to ~2×10^5 CFU/ml with refined MHB. Cell suspension (90 µl) was mixed with 10 µl of 10× stock solutions of HNP-1 or LL-37 in 96-well polypropylene microtiter plates (Cat. No. 3790; Costar) and incubated for 24 h at 37°C, without shaking. The next day, cell growth was visually inspected for determination of MIC.

Construction of SUMO-fusion protein overexpression plasmids. Plasmids pET-SUMO-AmiA, pET-SUMO-AmiB, pET-SUMO-AmiC, pET-SUMO-NlpD and pET-SUMO-EnvC were constructed as follows. A DNA fragment containing a gene coding for *amiA, amiB, amiC, nlpD* or *envC* but lacking a signal peptide region was amplified by PCR with wild-type *E. coli* MG1655 chromosomal DNA as template. Primers used in PCR for each gene are listed in Table S2. The amplified PCR fragments were purified and cloned into linearized TA-cloning plasmid pET-SUMO (Champion™ pET SUMO Expression System, ThermoFisher Scientific). Resulting plasmids (pET-SUMO-AmiA, pET-SUMO-AmiB, pET-SUMO-AmiC, pET-SUMO-NlpD, and pET-SUMO-EnvC) were verified by sequencing and transformed into a host strain *E. coli* BL21(DE3). In each of the overexpression plasmids, all proteins were overexpressed with a His_6-SUMO (H-SUMO) tag fused to their N-termini.

Purification of AmiA, AmiB, AmiC, NlpD, and EnvC proteins. *E. coli* strains carrying respective overexpression plasmids were grown in LB. Overnight cultures were diluted 200-fold into 300 ml of LB supplemented with kanamycin (50 µg/ml) and glucose (0.05%). After growing cultures to an OD_{600} of ~0.4 at 30°C, IPTG was added to a final concentration of 0.5 mM, and growth was continued for an additional 3-4 h. Cells were harvested by centrifugation, and cell pellets were stored at -80°C until use for protein purification. For purification, cell pellets were thawed on ice and resuspended in 15 ml of cold Buffer A (50 mM Tris-HCl, pH 8.0, 300 mM NaCl, 10% glycerol) containing 20 mM imidazole. The cells were disrupted by using French Press, and cell debris and membranes were removed by centrifugation at 10,000 × g for 20 min at 4°C. Fusion proteins in the soluble fraction were purified using the ProPur™ IMAC Kit (NUNC). Each protein was overexpressed and purified with a His_6-SUMO (H-SUMO) tag fused to its N-terminus. Following purification of an H-SUMO fusion protein, the H-SUMO tag was removed by using His_6-tagged SUMO protease (H-SP, Invitrogen). Cleavage reactions were passed through Ni-NTA resin to remove free H-SUMO and H-SP, yielding a pure preparation of the desired protein without added non-native amino acid residues. The only exception was AmiB where the extra sequence CSSG was left at its N-terminus (Fig. S4A).

Preparation of dye-labeled sacculi. Sacculi were prepared with cells of an *E. coli* BW25113 Δ*lpp* strain as described by Uehara *et al* (2, 3). Cells were grown overnight in LB, and diluted 100-fold in 1 L of LB. After growing cultures to OD_{600} ~0.5, cells were harvested by centrifugation and resuspended in 20 ml of PBS. The resulting cell suspension was added to 80 ml of boiling 5% SDS with vigorous stirring. The samples were boiled for 30 min, and incubated overnight at room temperature. The next day, sacculi were sedimented by ultracentrifugation at 100,000 × g for 1 h at 25°C. The pellets were washed three times with water, resuspended in 1

ml of water, mixed with α-amylase (a final concentration of 200 μg/ml, Sigma A6380), and incubated for 2 h at 37°C. After adding 110 µl of 10% SDS, the mixture was further incubated for 1 h at 95°C. Sacculi were pelleted by centrifugation at 100,000 × g for 30 min at room temperature and washed five times each with 1 ml of water. The final pellet was dissolved in 1 ml of water containing 0.02% sodium azide and stored at 4°C. To label with the dye Remazol Brilliant Blue R (RBB) (Sigma, R8001), 1 ml of the purified sacculi was mixed with 200 µl of 0.2 M RBB and 83 µl of 6 M NaOH. After adjusting the mixture volume to 10 ml with water, the mixture was incubated overnight at 37°C. The next day, 83 µl of 6 M HCl was added to neutralize pH and 200 µl of 10 × PBS. Labeled sacculi were pelleted (13,200 rpm × 30 min, room temperature) and washed with water until the supernatant become clear. Finally, the dye-labeled sacculi were resuspended in 2 ml water containing 0.2% azide, incubated for 3 h at 65°C to inactivate possibly contaminating lysozyme, and stored at 4°C.

Peptidoglycan degradation assay using the dye-labeled peptidoglycan. The assay was performed as described by Uehara *et al* (3), with a slight modification. Ten µl of RBB-labelled sacculi were incubated for 3 h at 37°C with purified amidases (AmiA, AmiB, or AmiC; a final concentration of ~3 µg/ml) and/or their cognate accessory proteins (EnvC or NlpD; a final concentration of ~3 µg/ml) in 50 µl of PBS buffer (10 mM Na_2HPO4, 2 mM KH_2PO4, 137 mM NaCl and 2.7 mM KCl, pH 7.4). Reactions were terminated by incubating them at 95°C for 5 min, followed by centrifugation at 21,000 × g for 20 min at room temperature. Supernatants were removed and their absorbance was measured at 595 nm using NanoDrop (Fig. S4C).

Antimicrobial peptide degradation assay. Purified amidases (AmiA, AmiB, or AmiC; a final concentration of ~3 µg/ml) and/or their cognate accessory proteins (EnvC or NlpD; a final concentration of ~3 µg/ml) were incubated with HNP-1 (1 µg) for 10 h at 37°C (Fig. S4D) or

LL-37 (1 µg; data not shown) in 20 µl of PBS buffer (10 mM Na_2HPO4, 2 mM KH_2PO4, 137 mM NaCl and 2.7 mM KCl, pH 7.4) with or without additional $CaCl_2$, $MgCl_2$, or $ZnCl_2$ at a final concentration of 1 mM. Reactions were terminated by adding 6 µl of 4 × sample loading buffer (12% SDS, 6% mercaptoethanol, 30% glycerol, 0.05% Coomassie blue G-250, 150 mM Tris/HCl , pH 7.0) and boiling for 5 min and were run on 16% Tricine-SDS PAGE (4) and visualized by Coomassie Brilliant Blue staining.

Transmission electron microscopy. Bacterial cells were grown overnight in 2 ml of LB, diluted 1:100 in 50 ml of LB, and grown to OD_{600} ~0.5. Cells were pelleted by centrifugation at 8,000 rpm in a Beckman Coulter Avanti J-E, washed three times with 0.1 M Sorensen phosphate buffer, and fixed with 1 ml 2.5% glutaraldehyde in 0.1 M Sorensen phosphate buffer. Dispersed/re-suspended cells were stored in the suspension at 4°C and transferred to the UIC electron microscopy facility on ice where cells were post-fixed in 1% osmium tetroxide, dehydrated in a series of ascending ethanol using propylene oxide as transitional solvent, and embedded and cured in Epoxy Resin Lx112. Thin cut sections (83 nm) were placed on 200 mesh copper standard grids and stained with 2% uranyl acetate and Reynolds lead citrate for viewing on Jeol Jem 1220 transmission electron microscope. Digital images were acquired with Gatan Erlangshen ES 10000 W Model 785 digital camera with digital micrograph software program 1.7.1 Digital Micrograph DM.

Lipopolysaccharide (LPS) purification and detection by immunoblot. For LPS preparation, cells were grown overnight in 2 ml of LD medium (5) (1% tryptone, 0.5% yeast extract, and 0.5% NaCl) at 37°C with shaking. The overnight culture was adjusted to OD_{600} ~0.5 for LPS extraction using the method described by Davis and Goldberg (6). For immunodetection of LPS, 10 µl of LPS samples were separated on a 12.5% Tris-Tricine PAGE and electro-transferred onto

0.45 micron nitrocellulose membrane (OSMONICS WP4HY00010) in semi-dry Bjerrum Schafer-Nielsen transfer buffer (Tris Base, 5.82 g/l; glycerine, 2.93 g/l; and 20% methanol) using BioRad Trans-Blot Turbo transfer system. The membrane was incubated overnight with a blocking buffer (1% casein, 20 mM Tris, 500 mM NaCl, and 0.05 % Tween 20) at 4°C, and further incubated for 1 h at 25°C with mouse monoclonal anti-LPS antibody diluted 1:100 (Hycult biotech). The membrane was then washed six times for 5 min each, incubated at room temperature for 1 h with fluorescein horse anti-mouse IgG antibodies (Vector laboratories) diluted 1:1000, and finally imaged using Typhoon scanner.

For detection of LPS in membrane fractions, the inner and outer membranes were prepared by sucrose gradient fractionation as described by Marani *et al*. (7), with a slight modification. Briefly, cells were grown overnight in LB, diluted 1:100 in 500 ml of LD broth and grown to OD_{600} ~1, and harvested. Cell pellets were resuspended in buffer K (50 mM triethanolamine, 250 mM sucrose, 1 mM EDTA, 1 mM dithiothreitol, 0.1 mg/ml Pefabloc at pH 7.5) and lysed twice using French Press. After removing cell debris by centrifugation, supernatant samples (~4 ml) were loaded per tube for 2-step sucrose separation (4 ml of 50% and 70% sucrose on bottom in 14 × 89 mm polyallomer tubes), and centrifuged for 14 h at 39,000 rpm in the SW41 rotor at 4°C. From the top of the tubes, 300 µl fractions were collected and stored at -20°C before use. Sixteen µl of each fraction was mixed with 2 × SDS sample loading buffer, run on a 12.5% Tris-Tricine PAGE (BioRad), and subsequently electro-transferred onto 0.45 micron nitrocellulose membrane as described above. LPS was detected using the same antibodies as described above. For quality control, NADH oxidase assays were performed as described by Reusch and Burger (8). Typically, the inner membrane fractions yielded NADH oxidase activity of 0.35-0.4 U/ml and the outer membrane fractions below 0.1 U/ml for both wild-type *E. coli* and Δ*envC* strains.

Outer and inner membrane protein profiles. Outer and inner membrane protein samples were prepared following the protocol from the laboratory of Dr. Robert E. W. Hancock (9). Wild-type *E. coli* and its isogenic Δ*envC* strains were grown to OD_{600} ~1.0 in 500 ml of LB. Cells were harvested by centrifugation (6,300 rpm × 20 min, at 4°C), and pellets were frozen and stored at -80°C until use. Each pellet was resuspended in 10.5 ml of cold lysis solution (10 mM Tris (pH 8.8) containing 20% sucrose, 50 µg/ml DNase I, and HALT protease inhibitor cocktail (Thermo Fisher Scientific) and incubated at room temp for 15 min. Cells were broken twice using French Press, and cell debris was removed by centrifugation. Supernatant samples (~4 ml) were loaded per tube for 2-step sucrose separation (4 ml of 50% and 70% sucrose on bottom in 14 × 89 mm polyallomer tubes), and centrifuged for 14 h at 39,000 rpm in the SW41 rotor at 4°C. Visible layers were collected (inner membrane layer is reddish/brown above 50% sucrose; outer membrane layer is white above 70% layer). For quality control, NADH oxidase activity was measured as described above. Collected samples were then concentrated by centrifugation in polyallomer centrifuge tubes (8.9 ml capacity) in Type 50 rotor at 49,600 rpm × 1 h, supernatants were removed, and final pellets were resuspended in Solubilizer 2 lysis buffer (Invitrogen) containing 2% CHAPS. Samples (~800 µg each in 10 µl) were mixed with 143.3 µl rehydration buffer used to rehydrate Zoom strips pH3-10NL (Invitrogen) overnight at 22°C and kept enclosed within a Styrofoam box containing a moist towel to prevent drying. Remaining steps were performed as recommended by the Invitrogen Zoom IEF manual, except that TCA precipitation step was omitted for membrane proteins (10). IEF strips were run on 1.0 mm × IPG NuPAGE Novex 4-12% Bis-Tris Zoom Gels (Invitrogen). Finally, proteins were visualized by Coomassie staining, and protein spots were excised and sent to UIC Protein Research Laboratory for identification by mass spectrometry.

Periplasmic protein profiles. The periplasmic protein samples were prepared by the chloroform release method (11), with a slight modification. Wild-type *E. coli* and its isogenic Δ*envC* mutant strains were grown to OD_{600} ~1.0 in 2 ml of LB, with shaking at 37°C. Cells were harvested by centrifugation (4,000 rpm × 10 min, at 4°C), most of the supernatants were removed, and cell pellets were resuspended in the residual supernatant. To release the periplasmic proteins, the cell resuspension was mixed with 20 µl of chloroform and vortexed, incubated at room temperature for 15 min, and mixed with 200 µl of buffer (10 mM Tris-HCl , pH 8.0). Finally, cells were removed by centrifugation (4,000 rpm × 20 min, at 25°C), and supernatants containing periplasmic proteins (200 µl) were transferred to eppendorf tubes. After determining protein concentrations using the Pierce BCA Protein Assay Kit, periplasmic protein samples (~20 µg each) were run on 4-20% Mini-PROTEAN® TGX (BioRad), and visualized with Coomassie Brilliant Blue staining.

Construction of a non-polar *E. coli* Δ*nlpD* mutant. The 3′ region of the *nlpD* gene contains promoter for the downstream gene *rpoS* (12). A non-polar Δ*nlpD* mutant was constructed using one-step inactivation method as described by Datsenko and Wanner (13) (see Table S1 and S2 in the Supplemental Materials for primers and strain information).

TABLE S1 Strains and plasmids used in this study

Species or Strain	Relevant genotype or characteristics	Source or reference
E. coli		
DH5α	Host strain used for cloning and plasmid preparation	Laboratory stock
JM109	Host strain used for cloning and plasmid preparation	Laboratory stock
BW25113	Parent strain, Δ(*araD-araB*)567 Δ*(rhaD-rhaB)568* Δ(*lacZ4787*::*rrnB*-3) *hsdR514 rph-1*	(13, 14)
BW25141	*lacI rrnB*$_{T14}$ Δ*lacZ*$_{WJ16}$ Δ*phoBR580 hsdR514* Δ*araBAD*$_{AH33}$ Δ*rhaBAD*$_{LD78}$ *galU95 endA*$_{BT333}$ *uidA*(Δ*Mlu*I)::*pir*$^{+}$ *recA1*	(13)
	Δ*phnB*::Km^{r}	(15)
	Δ*yhcB*::Km^{r}	(15)
	Δ*pheM*::Km^{r}	(15)
	Δ*nanA*::Km^{r}	(15)
	Δ*fimB*::Km^{r}	(15)
	Δ*yhcM*::Km^{r}	(15)
	Δ*amiB*::Km^{r}	(15)
	Δ*yjbS*::Km^{r}	(15)
	Δ*yjgX*::Km^{r}	(15)
	Δ*glpR*::Km^{r}	(15)
	Δ*mdh*::Km^{r}	(15)
	Δ*pdhR*::Km^{r}	(15)
	Δ*zapB (yiiU)*::Km^{r}	(15)
	Δ*envC (yibP)*::Km^{r}	(15)
	Δ*yhhN*::Km^{r}	(15)
	Δ*yadM*::Km^{r}	(15)
	Δ*yeeA*::Km^{r}	(15)
	Δ*JW5728*::Km^{r}	(15)
	Δ*metA*::Km^{r}	(15)
	Δ*basR*::Km^{r}	(15)
	Δ*yjgH*::Km^{r}	(15)
	Δ*yjdC*::Km^{r}	(15)
	Δ*purH*::Km^{r}	(15)
	Δ*yagF*::Km^{r}	(15)
	Δ*glyS*::Km^{r}	(15)
	Δ*yjgL*::Km^{r}	(15)
	Δ*yddJ*::Km^{r}	(15)
	Δ*yciN*::Km^{r}	(15)
	Δ*pgi*::Km^{r}	(15)
	Δ*yhdY*::Km^{r}	(15)
	Δ*nikD*::Km^{r}	(15)
	Δ*recO*::Km^{r}	(15)
	Δ*iclR*::Km^{r}	(15)
	Δ*yggR*::Km^{r}	(15)
	Δ*pntB*::Km^{r}	(15)

	Δ*atoA*::Km^r	(15)
	Δ*nhaA*::Km^r	(15)
	Δ*tatA*::Km^r	(15)
	Δ*phoP*::Km^r	(15)
	Δ*gpmM*:: Km^r	(15)
	Δ*yibQ*:: Km^r	(15)
	Δ*amiC*::Km^r	(15)
	Δ*hemF*::Km^r	(15)
	Δ*mutL*:: Km^r	(15)
	Δ*rpoS*:: Km^r	(15)
	Δ*envC*::frt	This study
	Δ*amiA682*:: Km^r	This study
	Δ*amiB856::* Km^r	This study
	Δ*nlpD449*:: Km^r	This study
	Δ*amiA682*::frt	This study
	Δ*amiB856::*frt	This study
	Δ*amiC::frt*	This study
	Δ*nlpD449*::frt	This study
	Δ*envC*::frt/p*envC*$_{Ec}$	This study
	Δ*yjgX*::Km^r/p*yjgX*$_{Ec}$	This study
S. enterica Typhimurium		
14028s	Parent strain	ATCC
	Δ*envC*::Cm^r	This study
	Δ*envC*::frt	This study
	Δ*phoP*::Tn*10* (MS7953s)	E. Groisman Lab
	Δ*pmrAB* (EG13937)	E. Groisman Lab
	Δ*rfaL*::Cm^r	This study
	Δ*rfbB*::Cm^r	This study
Plasmid		
pKD46	*araC bla oriR101 repA101*(ts) λ red (gam$^+$ bet$^+$ exo$^+$)	(13)
pKD3	oriRγ *bla* with the Cm^r cassette flanked by FRT sites	(13)
pKD4	oriRγ *bla* with the Km^r cassette flanked by FRT sites	(13)
pCP20	FLP (FRT-specific) recombinase, Amp^r Cm^r	(13)
pCL1920	pSC101-based with Spc^r	(16)
p*envC*$_{Ec}$	pCL1920 carrying a wild-type copy of the *E.coli envC* gene with its own upstream region	This study
p*zapB*$_{Ec}$	pCL1920 carrying a wild-type copy of the *E.coli zapB* gene with its own upstream region	This study
p*yjgX*$_{Ec}$	pCL1920 carrying a wild-type copy of the *E.coli yjgX* gene with its own upstream region	This study
pET-SUMO-AmiA	Overexpression of AmiA	This study
pET-SUMO-AmiB	Overexpression of AmiB	This study
pET-SUMO-AmiC	Overexpression of AmiC	This study

pET-SUMO-EnvC	Overexpression of EnvC	This study
pET-SUMO-NlpD	Overexpression of NlpD	This study

TABLE S2 Primers used in this study

Name	Sequence (5′ → 3′)	Purpose
pKD3 Cm F	CGCAAGGCGACAAGGTGCT	To check deletion mutants
pKD3 Cm R	CCAGCTGAACGGTCTGGTT	To check deletion mutants
pKD4 Km F	CCTCGTGCTTTACGGTATC	To check deletion mutants
pKD4 Cm R	CCTGCGTGCAATCCATCTT	To check deletion mutants
[a]Ec phnB del CK F	CGAACTACACTTAACTGGCT	To check deletion mutants
Ec yhcB del CK F	GGATTCCACTTCTGTGGAAT	To check deletion mutants
Ec pheM del CK F	GGAGCTAGTCTCCCTCTTTT	To check deletion mutants
Ec nanA del CK F	GCCACTTTAGTGAAGCAGAT	To check deletion mutants
Ec fimB del CK F	GCATGGCGTTTGTATGGCAA	To check deletion mutants
Ec yhcM del CK F	GACTGCATATCGCGTAGTGT	To check deletion mutants
Ec amiB del CK F	GGCCACAACAAGGTACAGG	To check deletion mutants
Ec yjbS del CK F	CCTCGGCAAATGAGCGAAAT	To check deletion mutants
Ec yjgX del CK F	CCTCTTGCAGTACCAGTGTA	To check deletion mutants
Ec glpR del CK F	GGTTTAGCGATGGCTTTTGT	To check deletion mutants
Ec mdh del CK F	GCAGCGGAGCAACATATCTT	To check deletion mutants
Ec pdhR del CK F	CCTGTATGGACATAAGGTGA	To check deletion mutants
Ec yiiU del CK F	GGTAATCGGGACGAGGATTT	To check deletion mutants
Ec yhhN del CK F	GCGACGACAATTAAGCCAAT	To check deletion mutants
Ec yadM del CK F	GCAGCAGGGAAATATCAGCA	To check deletion mutants

Ec yeeA del CK F	GGTGTGTTAACGCGATGAC	To check deletion mutants
Ec basR del CK F	CGGTATCAGGTTGACCAGAA	To check deletion mutants
Ec metA del CK F	CTTCAACATGCAGGCTCGA	To check deletion mutants
Ec yjdC del CK F	GGCCTACCGACAATTCTCTTT	To check deletion mutants
Ec purH del CK F	GAGCGTTGCGCAAACGTTTT	To check deletion mutants
Ec yagF del CK F	CGCAGATGTATCAGCTGGAT	To check deletion mutants
Ec glyS del CK F	GCCTACGAGCGTATTCTGAA	To check deletion mutants
Ec yjgL del CK F	CGTGATGACTTTTCGCCGTA	To check deletion mutants
Ec yddJ del CK F	CTGGGAAGAACAATTGCCCTA	To check deletion mutants
Ec yciN del CK F	GCGTCACACTGTACCTGATA	To check deletion mutants
Ec pgi del CK F	GCGCTAAGGGTTTACACTCA	To check deletion mutants
Ec yhdY del CK F	CGCTATTGGCTATCCCGATA	To check deletion mutants
Ec nikD del CK F	GCGCTGTTTATCAGCGTGAT	To check deletion mutants
Ec recO del CK F	GCGCGTAAAGACATGCAGGAAA	To check deletion mutants
Ec iclR del CK F	CCACCACGCAACATGAGATT	To check deletion mutants
Ec yggR del CK F	CTGACTGCAAGCAGCGTAAT	To check deletion mutants
Ec JW5728 del CK F	CGAAGAGCTACCTTTCACCT	To check deletion mutants
Ec nhaA del CK F	CGCTGATGGCGCAAATTCTT	To check deletion mutants
Ec gpmM del CK R	CTAAGAACGCTGGCGTAG	To check deletion mutants
Ec envC del CK R	GACGATGGCAAGTTTGCCA	To check deletion mutants

Ec phoP del CK F	GTTTACCCCCATAACCACATAAT	To check deletion mutants
pCL1920 seq F	GCTTCCGGCTCGTATGTTG	Cloning and Sequencing
pCL1920 seq R	GTGCTGCAAGGCGATTAAGT	Cloning and Sequencing
Ec envC npfwd HindIII	CTAGAAGCTTCAACTGCTGATCACCGCTG	Cloning into pCL1920
Ec envC rev BamHI	CTAGGGATCCACGACGAAATGGAAACAAAAC	Cloning into pCL1920
Ec envC rev2	GCTGACCTTGCTGGATGGA	Sequencing
Ec zapB npFwd HindIII	ATGAAGCTTGAGCGTTATCGCGCCATTTA	Cloning into pCL1920
Ec zapB EcoRI	GTCGAATTCTACCTGTTGGCCTACACAGT	Cloning into pCL1920
Ec amiA del F	gatataacatctggaactttattattacaactcaggccgtgtgtaggctggagctgcttc	*amiA682* deletion
Ec amiA del R	caccagcaccgaaggaaccgacggtgatttcaacaccacaatgggaattagccatggtcc	*amiA682* deletion
Ec amiA del CK F	aggctgattatggcgtgaac	To check deletion mutants
Ec amiA del CK R	acaccaccattacgcaacac	To check deletion mutants
Ec amiB del F	gctggcgcgtttagccggttaacctttgaaaggtggcggggtgtaggctggagctgcttc	*amiB856* deletion
Ec amiB del R	agccagctggccatttcactgttggcgcgacggttagagaatgggaattagccatggtcc	*amiB856* deletion
Ec amiB del CK F	attgttgctggcgcgtttag	To check deletion mutants
Ec amiB del CK R	ttctttgactaccgacgcag	To check deletion mutants
Ec nlpD del F	ttatgtcactggttattaaccaatttttcctgggggataagtgtaggctggagctgcttc	*nlpD449* deletion
Ec nlpD del R	caaggtctgaccaacgttcagcgcgtatggtgcctgaataatgggaattagccatggtcc	*nlpD449* deletion
Ec nlpD del CK F	tgcgctttgtccctttagtg	To check deletion mutants
Ec nlpD del CK R	agttcctcttcggccaaatc	To check deletion mutants
Ec amiA SUMO F	gccatcgccaaagacgaac	amiA cloning into pET-SUMO
Ec amiA SUMO R	ctgtttaacctggtgtgcgt	amiA cloning into pET-SUMO
Ec amiB SUMO F	tgctcttccggtgcgacgctctctgatattca	amiB cloning into pET-SUMO

Ec amiB SUMO R	gtaagacctgaattggcatc	amiB cloning into pET-SUMO
Ec envC SUMO F	gatgagcgtgaccaactcaa	envC cloning into pET-SUMO
Ec envC SUMO R	cgttacgacgaaatggaaac	envC cloning into pET-SUMO
Ec amiC SUMO F	gcggtcagccaggtcgtg	amiC cloning into pET-SUMO
Ec amiC SUMO R	cgccattcagcgccttttta	amiC cloning into pET-SUMO
Ec nlpD SUMO F	tctgacacttcaaatccacc	nlpD cloning into pET-SUMO
Ec nlpD SUMO R	cttgacggaacattcaagca	nlpD cloning into pET-SUMO
pET-SUMO F	caagctgatcagacccctga	Sequencing pET-SUMO plasmids
pET-SUMO R	gcagccggatctcagtggt	Sequencing pET-SUMO plasmids
[b]St envC del F	gactggtaagccgctgttcatcgtggaataatccctccccgtgtaggctggagctgcttc	*envC* deletion
St envC del R	gccttgtagccagtccgccagaatcaccctgccgtcggcgatgggaattagccatggtcc	*envC* deletion
St envC del CK F	CAGACATTGCGCCTACCATG	To check deletion mutants
St rfaL del F	agagactctgtctcatcccaaacctattgtggagaaaaggtgtaggctggagctgcttc	*rfaL* deletion
St rfaL del R	gaaaacgcgctgataccgtaataagtatcagcgcgtttttatgggaattagccatggtcc	*rfaL* deletion
St rfaL del CK F	ccacaagcgtatttggaaag	To check deletion mutants
St rfaL del CK R	gtgattgagtcctgatgatg	
St rfbB del F	aattaaataccacattaaatacgccttatggaatagaaaagtgtaggctggagctgcttc	*rfbB* deletion
St rfbB del R	caactctgatacgccccacttttaacattgtttacccattatgggaattagccatggtcc	*rfbB* deletion
St rfbB del CK F	gtgtaacgacttgagcaatt	To check deletion mutants
St rfbB del CK R	ccttcatagttctgttctat	

[a]Ec indicates *E. coli* BW25113 and [b]St *Salmonella enterica* Typhimurium 14028s.

TABLE S3 Mutants identified as putatively hypersusceptible or hyper-resistant to HNP-1

Mutants putatively identified as being hypersusceptible to HNP-1				
No.	Fold Change (Control/HNP-1)	Gene name	JW annotation in W3110	b annotation in K-12
1	9.20	*phnB*	JW4068	b4107
2	7.80	*yhcB*	JW5539	b3233
3	7.23	*pheM*	JW1705	b1715
4	6.75	*nanA*	JW3194	b3225
5	6.60	*fimB*	JW4275	b4312
6	6.28	*yhcM*	JW3201	b3232
7	5.84	*amiB*	JW4127	b4169
8	5.31	*yjbS*	JW5717	b4054
9	5.13	*yjgX*	JW5763	No b annotation; located inside *yjgW* (b4274)
10	4.50	*glpR*	JW3386	b3423
11	4.44	*mdh*	JW3205	b3236
12	4.40	*pdhR*	JW0109	b0113
13	4.36	*yiiU (zapB)*	JW3899	b3928
14	3.92	*yibP (envC)*	JW5646	b3613
15	3.85	*yhhN*	JW3433	b3428
16	3.54	*yadM*	JW0134	b0138
17	3.48	*yeeA*	JW1990	b2008
18	3.21	*JW5728*	JW5728	No b annotation
19	3.13	*metA*	JW3973	b4013
20	3.04	*basR*	JW4074	b4113
21	2.65	*yjgH*	JW4206	b4248
22	2.51	*yjdC*	JW5733	b4135
23	2.47	*purH*	JW3970	b4006
24	2.37	*yagF*	JW0262	b0269
25	2.31	*glyS*	JW3530	b3559
26	2.26	*yjgL*	JW5757	b4253
27	2.26	*yddJ*	JW1466	b1470
28	2.20	*yciN*	JW1265	b1273
29	2.13	*pgi*	JW3985	b4025
30	2.12	*yhdY*	JW5545	b3270
31	2.05	*nikD*	JW3444	b3479
32	2.04	*recO*	JW2549	b2565
33	2.03	*iclR*	JW3978	b4018
34	2.03	*yggR*	JW2917	b2950
Mutants putatively identified as being hyper-resistant to HNP-1				
No.	Fold Change (Control/HNP-1)	Gene name	JW annotation in W3110	b annotation in K-12
1	0.0933	*pntB*	JW1594	b1602
2	0.0758	*atoA*	JW2216	b2222

3	0.0712	*nhaA*	JW0018	b0019
4	0.0683	*cysB*	JW1267	b1275
5	0.0603	*tatA*	JW3813	b3836

TABLE S4 MICs of HNP-1 and LL-37 against the Δ*envC* mutant

	MIC[a] (µg/ml)	
Strain	HNP-1	LL-37
WT$_{Ec}$	>250	7.8
Δ*envC*	62.5	3.9
Δ*envC*/p*envC*	>250	7.8

[a]MIC was determined as described in the supplemental methods. Shown is the mean of values obtained from two independent experiments performed in duplicate, which gave identical results.

TABLE S5 Proteins whose expression level is altered in the Δ*envC* mutant relative to WT_{Ec}

Spot No.[a]	Relative changes in Δ*envC* vs. WT_{Ec}	Protein name	pI	Description/Function
From outer membrane protein samples run on 2-DE gel				
1	Down	OmpA	5.99	Outer membrane porin A
	Down	TolC	5.46	Outer membrane channel protein
	Down	OmpT	5.76	Outer membrane protease
2	Down	FliC	4.5	Flagella filament structural protein (flagellin)
	Down	OmpC	4.39	Outer membrane porin C
	Down	LamB	4.62	Maltose outer membrane porin
	Down	OmpF	4.57	Outer membrane porin F
From inner membrane protein samples run on 2-DE gel				
3	Up	AcrB	5.15	Acriflavin resistance protein B
	Up	AdhE	6.32	Pyruvate-formate-lyase deactivase
	Up	NuoG	5.85	NADH:ubiquinone oxidoreductase subunit G
4	Down	HflC	6.29	Inner membrane protein that regulates FtsH
	Down	EmrA	9.33	Inner membrane protein for the EmrAB efflux pump

[a]See numbering in the 2-DE gel images in Fig. S7 in the Supplemental Information.

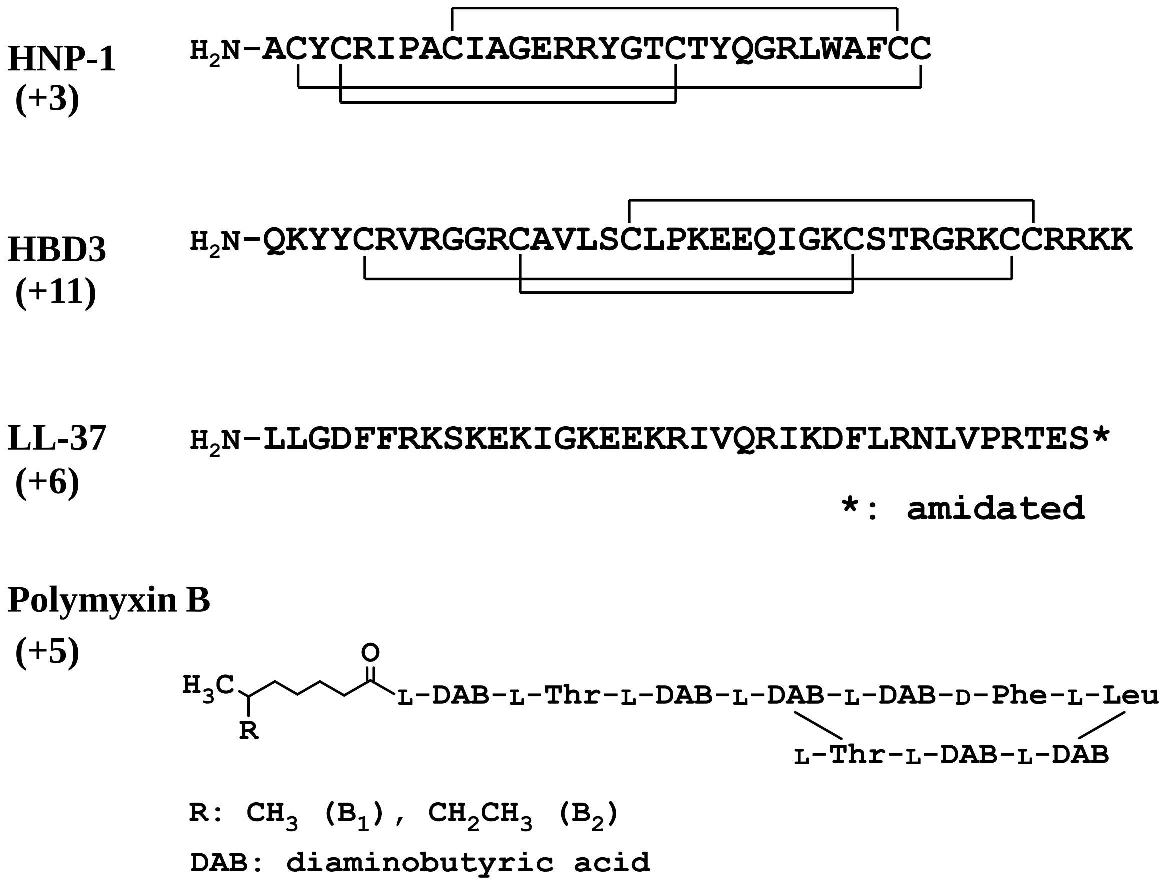

FIG S1 Cationic antimicrobial peptides used in this study. Numbers in parenthesis indicate the positive charges of respective peptides. β-sheet peptide: HNP-1 and HBD3; α-helical peptide: LL-37; and cyclic lipopeptide: polymyxin B

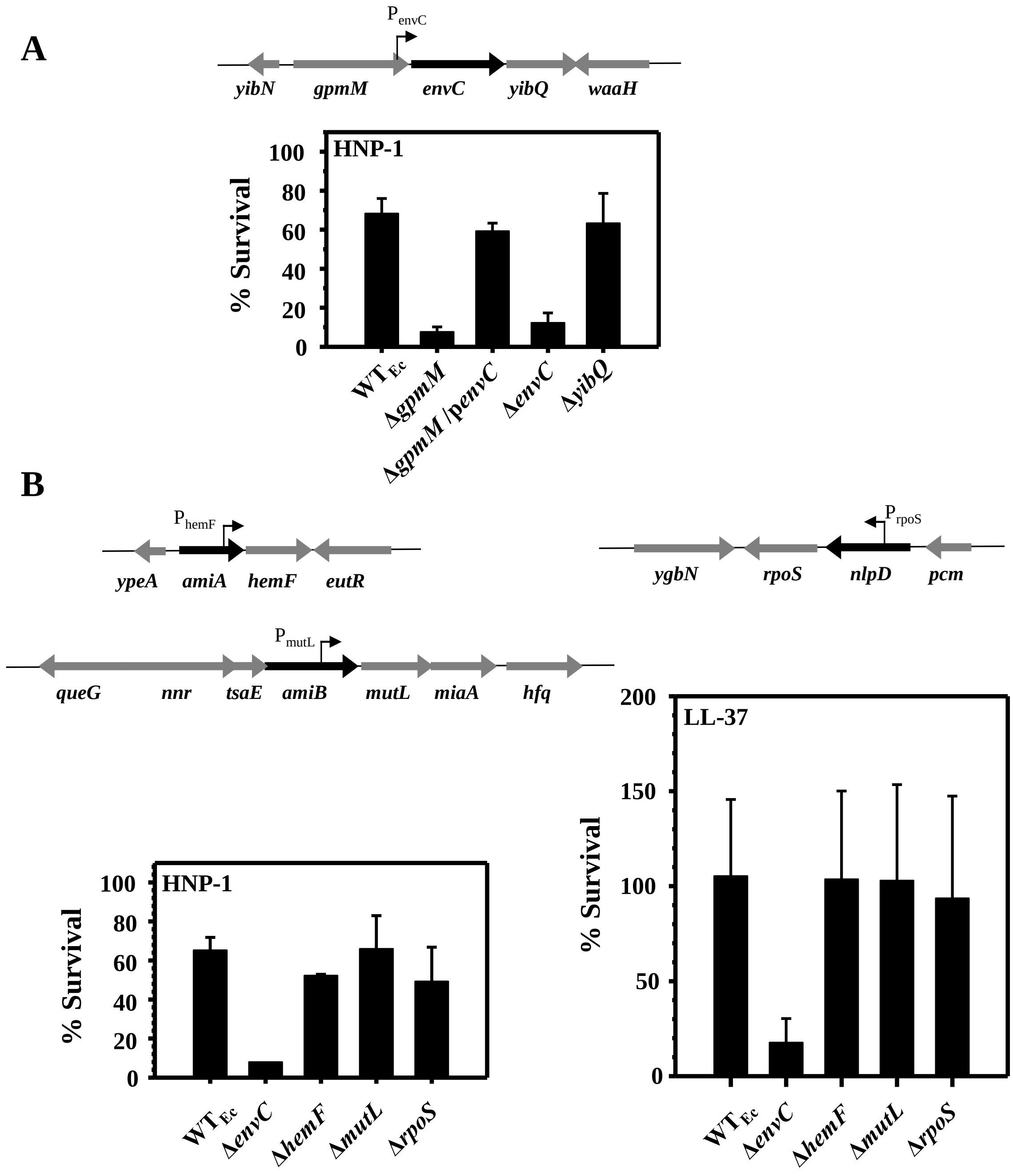

FIG S2 The neighboring genes of *envC*, *amiA*, *amiB*, and *nlpD* are not involved in CAMP resistance. (A) Genetic organization of neighboring genes *of envC* in *E. coli*. (B) Genetic organization of neighboring genes of *amiA*, *amiB*, and *nlpD* in *E. coli*. Mutants deleted for the downstream gene of *amiA*, *amiB*, *envC*, or *nlpD* show wild-type susceptibility to both HNP-1 and LL-37. Wild-type *E. coli* and mutant strains grown to mid-log phase in LB were examined for HNP-1 (100 µg/ml) or LL-37 (5 µg/ml) susceptibility.

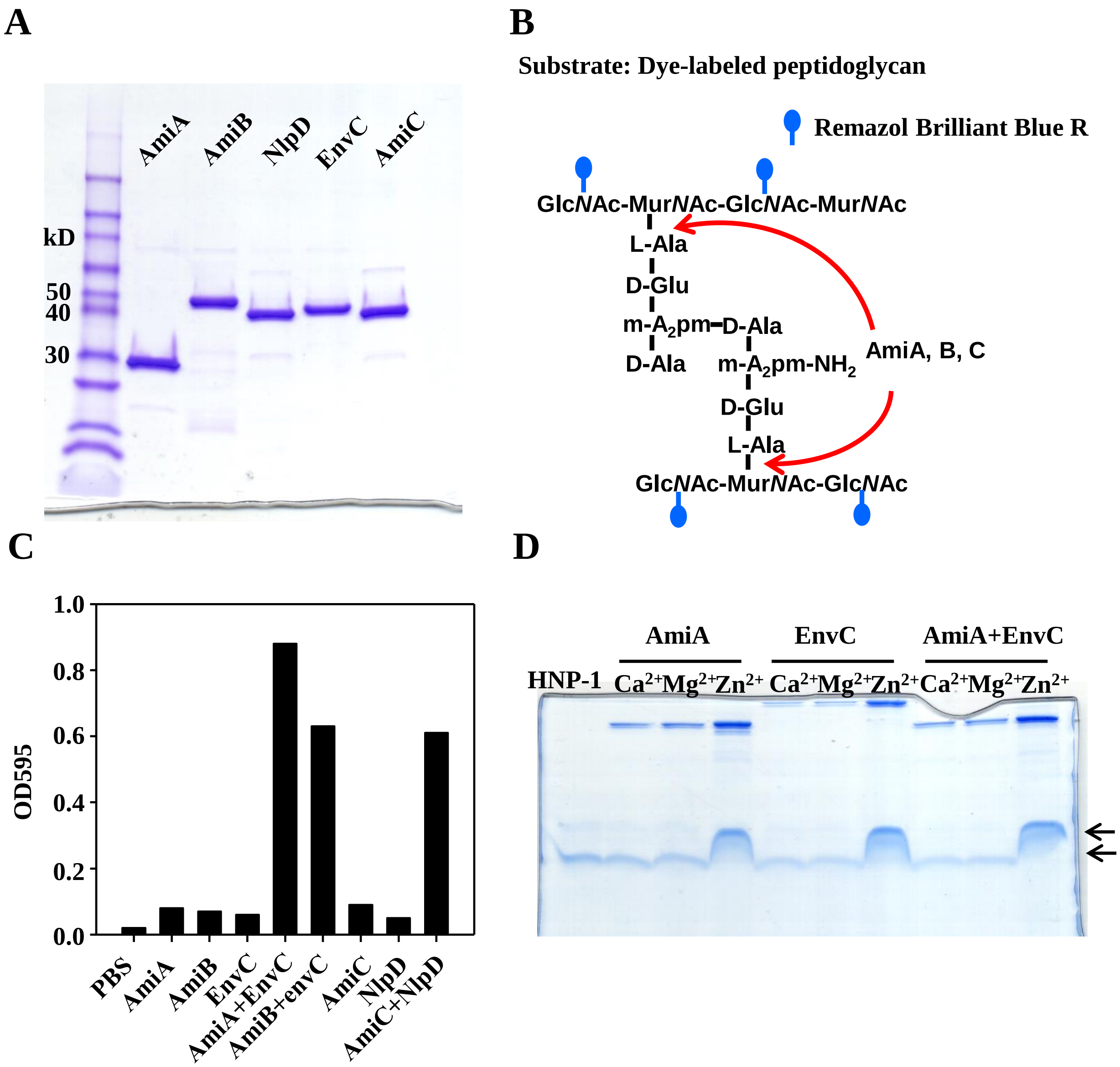

FIG S3 EnvC and its cognate amidases do not degrade HNP-1. (A) Purified proteins were run on 4–20% SDS-polyacrylamide gel and stained with Coomassie Blue. (B) Dye-labeled peptidoglycan. (C) Cleavage of peptidoglycan by amidases (EnvC+AmiA; EnvC+AmiB; and NlpD+AmiC). (D) HNP-1 degradation assay (see Supplemental Methods for details). Arrows indicate intact HNP-1. The amidase (AmiB+EnvC) did not degrade HNP-1 (data not shown), and none of the amidases, (AmiA+EnvC), (AmiB+EnvC), and (AmiC+NlpD), cleaved LL-37 (data not shown).

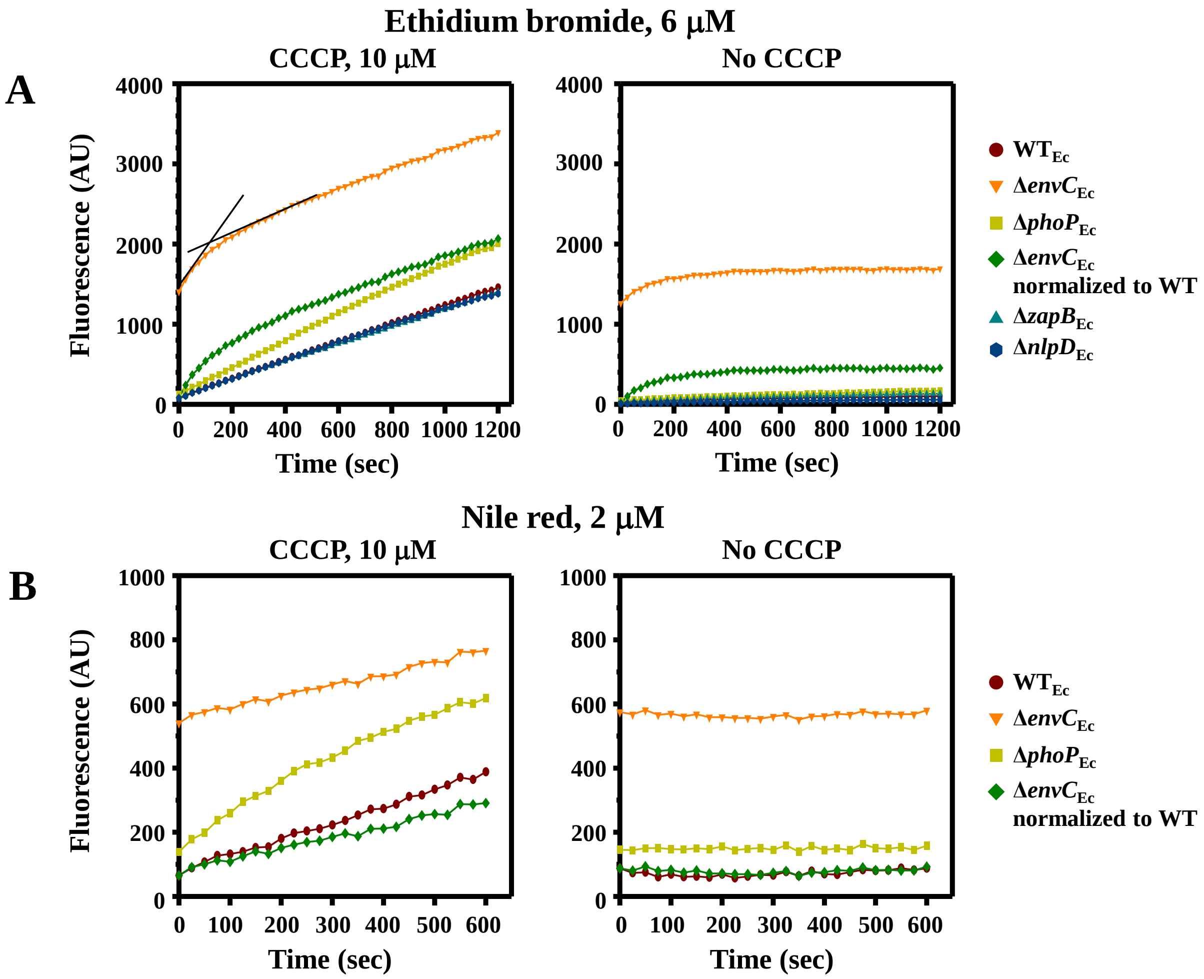

FIG S4 The *E. coli* Δ*envC* mutant exhibits altered outer membrane permeability. Outer membrane permeability was determined by measuring the rate of fluorescent dye uptake using (A) Ethidium bromide and (B) Nile red. Values for "Δ*envC*$_{Ec}$ normalized to WT" were obtained by subtracting Δfluorescence$_{t=0}$ (WT$_{Ec}$−Δ*envC*$_{Ec}$, at zero time point) from values of Δ*envC*$_{Ec}$ at other time points. Note that the Δ*envC*$_{Ec}$ mutant exhibits bi-phasic uptake of ethidium bromide. Shown is a representative of more than three independent experiments with similar trends.

Wild-type *E. coli* **Δ*envC*$_{Ec}$**

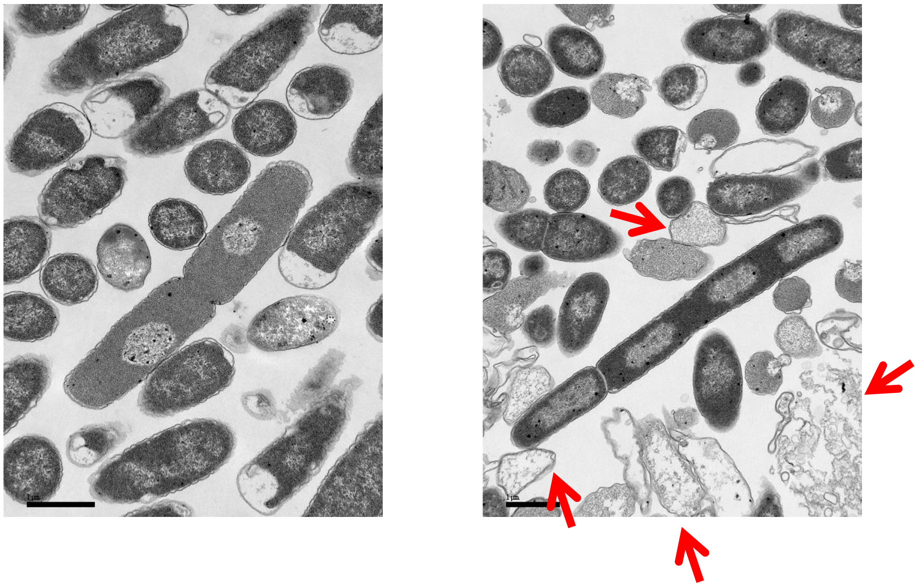

FIG S5 Microscopic examination of the *E. coli* wild-type and Δ*envC* strains. Transmission electron micrograph of wild-type *E. coli* and Δ*envC*$_{Ec}$ mutant strains. Wild-type *E. coli*, × 28100 magnification and Δ*envC*$_{Ec}$, × 22000 magnification. Red arrows indicate ghost cells and cell debris, which are not observed in the wild-type.

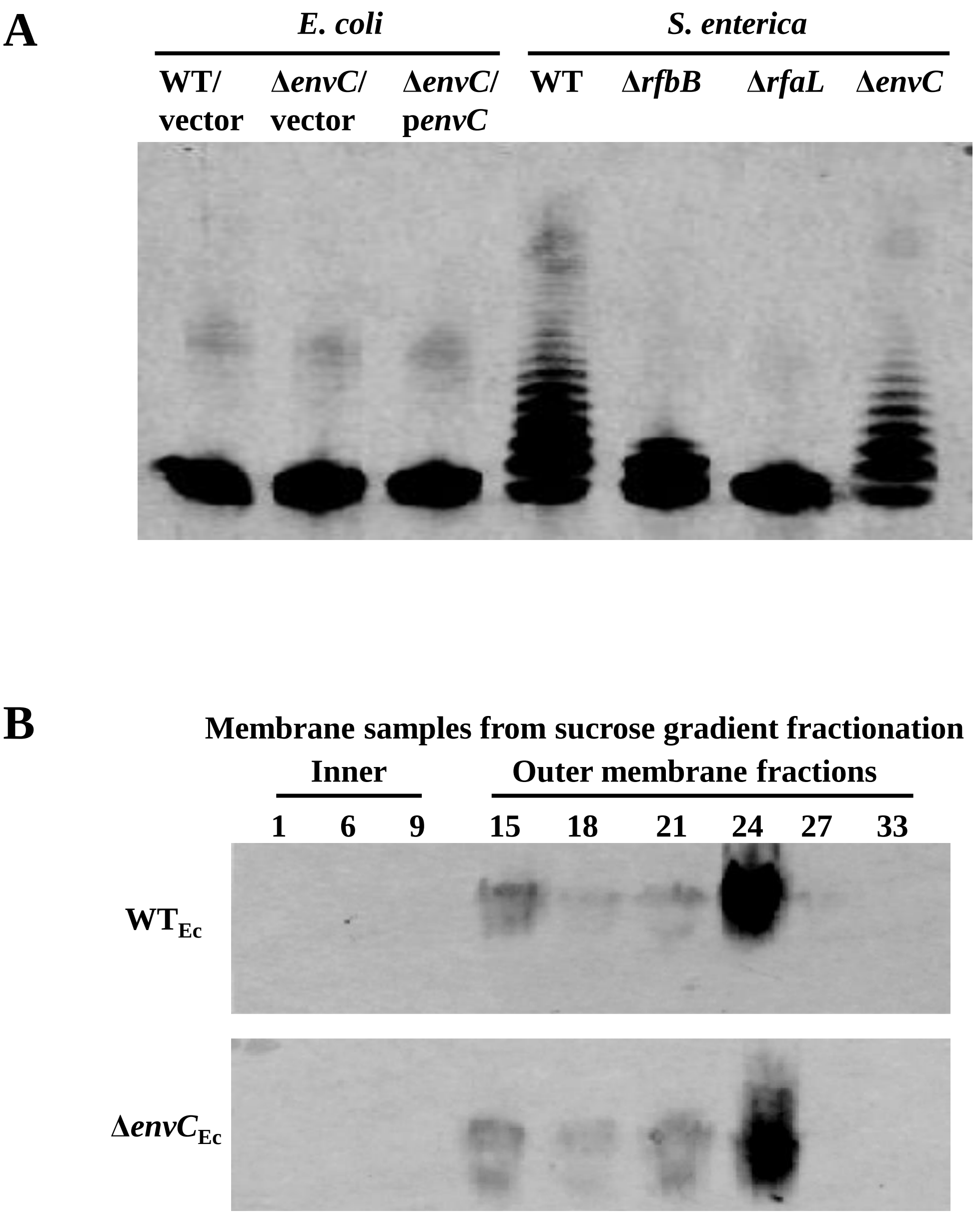

FIG S6 Inactivation of *envC* does not alter the length and distribution of LPS. (A) Western blot of lipid A for *E. coli* (BW25113) and *S. typhimurium* (14028s). *Salmonella* Δ*rfaL* and Δ*rfbB* mutants produce truncated LPS and are used as controls. (B) Western blot of lipid A in membrane fractions prepared by sucrose gradient fractionation: the inner membrane fractions (5-11) and outer membrane fractions (12-25).

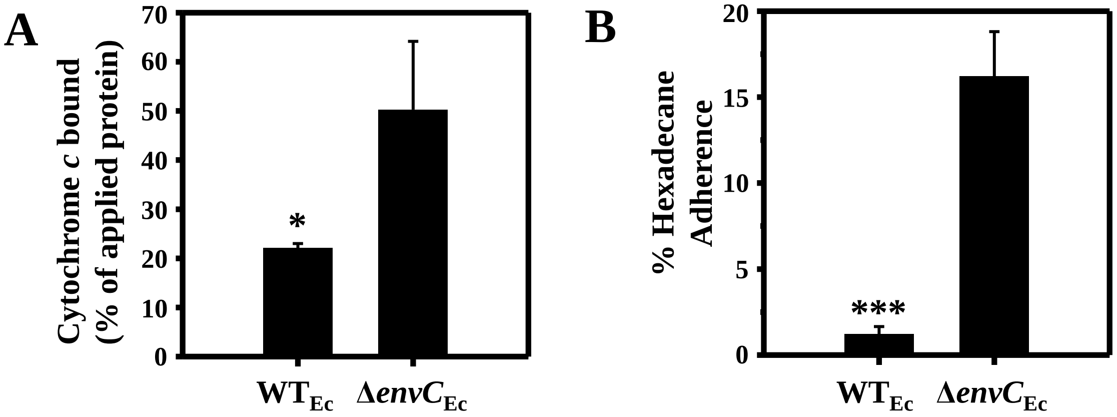

FIG S7 The *E. coli* Δ*envC* mutant exhibits altered surface properties. (A) Surface charges was measured via binding of the cationic protein cytochrome *c* to cells and represented as the percent bound mean±s.d. of three independent experiments performed in duplicate. (B) Hydrophobicity of cell surfaces was determined as adherence of cells to hexadecane and represented as the mean±s.d. of three independent experiments. Statistical significance was determined by paired, two-tailed Student's *t*-test (*, $p<0.05$ and ***, $p<0.001$).

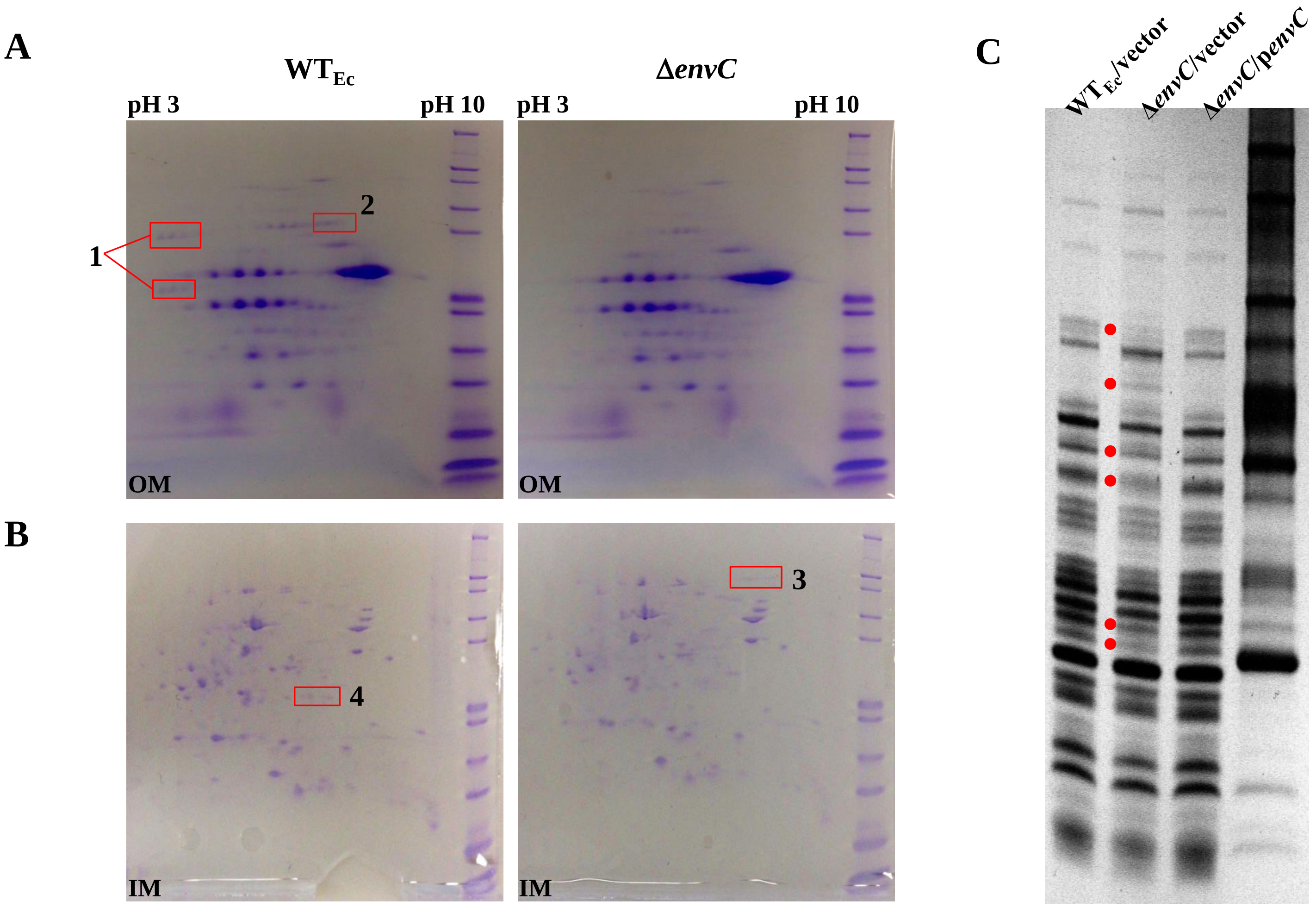

FIG S8 Membrane and periplasmic protein profiles of the *E. coli* wild-type and Δ*envC* strains. (A) Outer membrane (OM) protein samples (800 µg each) run on 2-DE gels. (B) Inner membrane (IM) protein samples (800 µg each). Protein spots in boxed area in 2-DE gels were excised and analyzed by mass spectrometry. Note that #1 is a combined sample. See Supplemental Methods for 2-DE procedures and Table S5 for the list of proteins identified by mass spectrometry. (C) Periplasmic protein samples (20 µg each) were run on 4-20% TGX gel (Bio-rad) and visualized by Coomassie staining. Red dots indicate protein bands with pronounced changes in intensity and restored to wild-type levels upon genetic complementation.

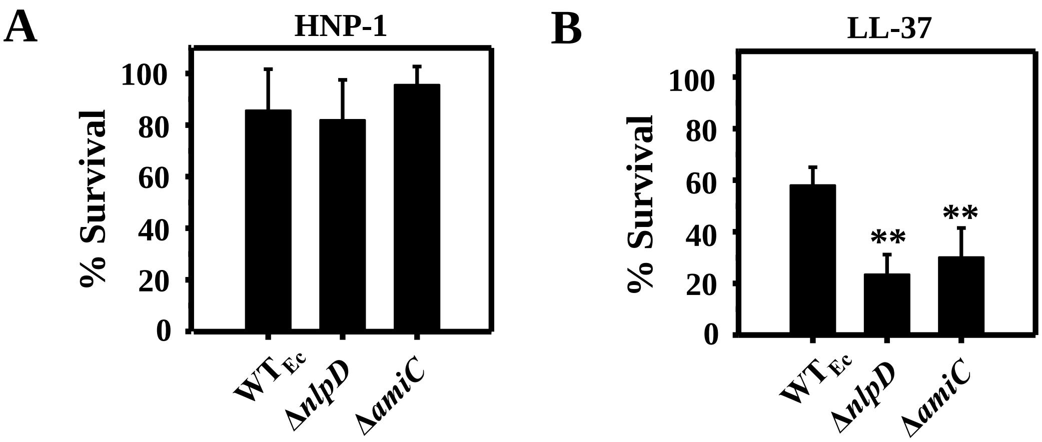

FIG S9 The *E. coli* Δ*nlpD* and Δ*amiC* mutants are hypersusceptible to LL-37, but not to HNP-1. (A) HNP-1 (at 100 µg/ml) killing assay. (B) LL-37 (at 5 µg/ml) killing assay. A non-polar Δ*nlpD* mutant was constructed (Table S1 and S2), and a mutant deleted for *rpoS*, the downstream gene of *nlpD*, was confirmed for wild-type HNP-1 and LL-37 susceptibilities (Fig. S2B). Killing assays for Δ*nlpD* and Δ*amiC* strains were performed at the same time with other strains shown in Fig. 3. Shown is the mean±s.d. of three independent experiments. Statistical significance was determined by one-way ANOVA test (**, p<0.01).

Supplemental References

1. **Turner J, Cho Y, Dinh N-N, Waring AJ, Lehrer RI.** 1998. Activities of LL-37, a cathelin-associated antimicrobial peptide of human neutrophils. Antimicrob Agents Chemother **42:**2206-2214.
2. **Uehara T, Dinh T, Bernhardt TG.** 2009. LytM-domain factors are required for daughter cell separation and rapid ampicillin-induced lysis in *Escherichia coli*. J Bacteriol **191:**5094-5107.
3. **Uehara T, Parzych KR, Dinh T, Bernhardt TG.** 2010. Daughter cell separation is controlled by cytokinetic ring-activated cell wall hydrolysis. EMBO J **29:**1412-1422.
4. **Thomassin J-L, Lee MJ, Brannon JR, Sheppard DC, Gruenheid S, Le Moual H.** 2013. Both group 4 capsule and lipopolysaccharide O-antigen contribute to enteropathogenic *Escherichia coli* resistance to human α-defensin 5. PLoS ONE **8:**e82475.
5. **Sperandeo P, Lau FK, Carpentieri A, De Castro C, Molinaro A, Dehò G, Silhavy TJ, Polissi A.** 2008. Functional analysis of the protein machinery required for transport of lipopolysaccharide to the outer membrane of *Escherichia coli*. J Bacteriol **190:**4460-4469.
6. **Davis JMR, Goldberg JB.** 2012. Purification and visualization of lipopolysaccharide from Gram-negative bacteria by hot aqueous-phenol extraction. J Vis Exp **28:**e3916.
7. **Marani P, Wagner S, Baars L, Genevaux P, De Gier J-W, Nilsson I, Casadio R, Von Heijne G.** 2006. New *Escherichia coli* outer membrane proteins identified through prediction and experimental verification. Protein Sci **15:**884-889.

8. **Reusch VM, Burger MM.** 1974. Distribution of marker enzymes between mesosomal and protoplast membranes. J Biol Chem **249:**5337-5345.

9. **Yakhnina AA, McManus HR, Bernhardt TG.** 2015. The cell wall amidase AmiB is essential for *Pseudomonas aeruginosa* cell division, drug resistance and viability. Mol Microbiol **97:**957-973.

10. **Sato T, Ito K, Yura T.** 1977. Membrane proteins of *Escherichia coli* K-12: Two-dimensional polyacrylamide gel electrophoresis of inner and outer membranes. Eur J Biochem **78:**557-567.

11. **Ames GF, Prody C, Kustu S.** 1984. Simple, rapid, and quantitative release of periplasmic proteins by chloroform. J Bacteriol **160:**1181-1183.

12. **Lange R, Fischer D, Hengge-Aronis R.** 1995. Identification of transcriptional start sites and the role of ppGpp in the expression of *rpoS*, the structural gene for the sigma S subunit of RNA polymerase in *Escherichia coli*. J Bacteriol **177:**4676-4680.

13. **Datsenko KA, Wanner BL.** 2000. One-step inactivation of chromosomal genes in *Escherichia coli* K-12 using PCR products. Proc Natl Acad Sci U S A **97:**6640-6645.

14. **Grenier F, Matteau D, Baby V, Rodrigue S.** 2014. Complete genome sequence of *Escherichia coli* BW25113. Genome Announc **2:**e01038-01014.

15. **Baba T, Ara T, Hasegawa M, Takai Y, Okumura Y, Baba M, Datsenko KA, Tomita M, Wanner BL, Mori H.** 2006. Construction of *Escherichia coli* K-12 in-frame, single-gene knockout mutants: the Keio collection. Mol Syst Biol **2:**2006.0008.

16. **Lerner CG, Inouye M.** 1990. Low copy number plasmids for regulated low-level expression of cloned genes in *Escherichia coli* with blue/white insert screening capability. Nucleic Acids Res **18:**4631.
